# CRISPR/Cas12a-based method coupled with isothermal amplification to identify *Alternaria* spp. isolated from wheat grain samples

**DOI:** 10.3389/fmicb.2024.1468336

**Published:** 2025-01-15

**Authors:** Aisha Shaizadinova, Meruyert Amanzholova, Irina Rukavitsina, Sailau Abeldenov, Anuar Rysbekovich Zhumakayev

**Affiliations:** ^1^Laboratory of Molecular Biotechnology, National Center for Biotechnology, Astana, Kazakhstan; ^2^Faculty of Biology and Biotechnology, Al-Farabi Kazakh National University, Almaty, Kazakhstan; ^3^Laboratory of Microbiology, A.I. Barayev Research and Production Centre for Grain Farming, Shortandy-1, Kazakhstan

**Keywords:** *Alternaria* detection, wheat diseases, CRISPR diagnostics, Cas12a, fungal plant pathogens

## Abstract

*Alternaria* fungal species are considered major plant pathogens, infecting various crops and resulting in significant agricultural losses. Additionally, these species can contaminate grain with multiple mycotoxins that are harmful to humans and animals. Efficient pest management relies on timely detection and identification of phytopathogens in plant and grain samples, facilitating prompt selection of a crop protection strategy. Conventional identification tools, such as morphological characterization and identification based on polymerase chain reaction (PCR)-based methods, are time-consuming and laboratory-bound, limiting their implementation for on-site diagnostics essential in the agricultural industry. Isothermal amplification methods, including nucleic acid sequence-based amplification (NASBA), loop-mediated isothermal amplification (LAMP), and recombinase polymerase amplification (RPA), enable nucleic acid amplification at constant temperatures, making them ideal for point-of-care diagnostics without the need for thermal cycling equipment. Clustered regularly interspaced short palindromic repeats (CRISPR)/CRISPR-associated protein 12a (Cas12a)-based identification, coupled with such isothermal amplification methods, represents an emerging nucleic acid-based technology for detecting plant pathogens at high accuracy and sensitivity. This study aimed to develop a CRISPR/Cas12a-based method integrated with RPA amplification for specific detection of *Alternaria* spp. isolated from wheat grain samples. The developed method targeted the β-tubulin gene was successfully identified *Alternaria* strains within a 20-min RPA amplification followed by a 30-min CRISPR/Cas12a reaction and visualization of results. Specificity test included pathogenic fungal species commonly hosted wheat grain, such as *Fusarium* spp. *Bipolaris sorokiniana*, and *Nigrospora oryzae* revealed high specificity of the method for *Alternaria* species. Furthermore, the method exhibited high sensitivity, detecting *Alternaria* DNA down to 100 copies, validated by real-time fluorescence readout. A fluorescence assay was employed to visualize the results of RPA and CRISPR/Cas12a reaction, demonstrating substantial implementation potential of the method in point-of-care detection of *Alternaria* spp. In conclusion, we present the CRISPR/Cas12a-based method as a potentially sustainable approach for the rapid, precise, and specific nucleic-acid-based identification of *Alternaria* species in grain samples.

## 1 Introduction

Ensuring food security is among the most crucial challenges at both international and national scales, recognized under the 17 Sustainable Development Goals (SDGs) adopted by the United Nations (2015). According to the Food and Agricultural Organization (FAO), the human population will exceed 9 billion by 2050, which will require an increase in cereal production up to 3.0 billion tons per annum (FAO, 2009). This challenge can be addressed by applying complex measures, including effective management of plant pathogens as one of the significant yield-decreasing factors. A variety of phytopathogens, including more than 19,000 plant fungal pathogens, can cause substantial quantitative and qualitative crop losses resulting in crop losses at 20–40%, economic declines at 220 billion dollars, and food poisoning by produced mycotoxins (Jain et al., 2019; Mitra, 2021; Francesconi, 2022). For example, wheat, one of the most important global crops, is susceptible to infection by 25 fungi, 3 bacteria, 1 virus, and 3 nematodes. At the same time, four and eight additional diseases are attributed to physiological-genetic and abiotic stress factors, respectively (Koyshibaev, 2018). Among different pathogenic fungi, *Alternaria* is considered the most prevalent mycotoxigenic fungal genus frequently hosted cereals crops worldwide, with a high incidence rate in grains of up to 90% (Pinto and Patriarca, 2017). *Alternaria* also represents a serious challenge due to its species harming a variety of crops with different diseases, including *Alternaria*-associated black point and leaf blight diseases (Tralamazza et al., 2018; Somma et al., 2019; Turzhanova et al., 2020). Furthermore, species of this genus are capable of producing more than 70 toxins while the most frequently detected mycotoxins harmful to humans and animals include alternariol (AOH), alternariol monomethyl ether (AME), tenuazonic acid (TA), and altertoxins (ATX) (Lou et al., 2013; Tralamazza et al., 2018; Wang et al., 2022; Castañares et al., 2023).

Such vast diversity of plant pathogenic species, the widespread impact of *Alternaria* on crop health, and the associated risks to food security emphasizes the complexity of pest management strategies for controlling *Alternaria* outbreaks efficiently to prevent significant yield losses and minimize mycotoxin contamination. An efficient crop protection strategy requires an initial yet crucial step as early detection and robust identification of phytopathogens on site, which is considered the primary objective in diagnostics (Aslam et al., 2017). Traditional identification methods include visual inspection of the infected plant or isolation with subsequent incubation and microscopic identification, usually coupled with biochemical assays. However, these methods require substantial time for fungal incubation and growth and are highly dependent on the skills of specialists and their knowledge of diverse fungal morphology and constantly updating taxonomy (Zhao et al., 2014; Chen et al., 2023). The application of immunofluorescence assays developed to detect a variety of plant pathogens, and mycotoxins is limited due to their weak sensitivity, affinity, and vulnerability to contaminants, alongside high fungi variability and phenotypic serological plasticity (Hariharan and Prasannath, 2021). Currently, molecular approaches based on widely applied polymerase chain reaction (PCR) are considered the most sophisticated and robust tools for pathogen detection, identification, and quantification (Aslam et al., 2017). Apart from intensively used internal transcribed spacer 1 (ITS1)/ITS4 classic primers (White et al., 1990), numerous PCR protocols targeting different genetic loci were developed for a wide range of plant fungal pathogens (McCartney et al., 2003; Kuzdraliński et al., 2017). Nevertheless, the PCR method has several drawbacks, including needing a specialized laboratory with sophisticated equipment, highly-trained personnel, and expensive reagents. Also, PCR methods are not portable for field conditions, which is crucial as crop production usually demands identification methods directly in the field (Ray et al., 2017; Chen Q. et al., 2024). Therefore, while molecular-based diagnostic methods have been advanced remarkably, further substantial research studies are essential to enhance their efficacy and accessibility in crop disease diagnostic systems (Aslam et al., 2017; Jain et al., 2019; Hariharan and Prasannath, 2021; Mitra, 2021).

Clustered regularly interspaced short palindromic repeats (CRISPR)-based diagnostics is considered as of the currently promising nucleic acid-based technologies to detect various agents with high accuracy, sensitivity, and in a prompt manner (Li et al., 2018b; Kaminski et al., 2021; Qiu et al., 2022). The key player in this system is the CRISPR-associated protein 12a (Cas12a) endonuclease, discovered in 2015 (Zetsche et al., 2015). Several unique traits characterize Cas12a: it operates with only one guide RNA (gRNA, also called crRNA), uses the protospacer adjacent motif (PAM) region (known as TTTN sequence) to bind crRNA to the target sequence, and forms sticky-end double-strand hydrolysis of DNA. In addition to these characteristics that facilitate the implementation of Cas12a in diagnostics, Cas12a possesses a specific trait: after binding to the target sequence, the enzyme undergoes conformational changes and begins to cleave any nonspecific single-stranded DNA (Chen et al., 2018; Li et al., 2018a). This activity, known as collateral or *trans*-cleavage activity, is crucial in diagnostic applications of the CRISPR/Cas12a method (Li et al., 2018b). The addition of fluorescently labeled single-stranded DNA (reporter with a quencher) to the reaction will result in either the cleavage of it by Cas12a and subsequent fluorescence signal (if a tested sample is positive for target genetic locus) or no cleavage and no consequent fluorescence activity (if a tested sample is negative and no conformational changes of Cas12a and its trans-activity occurred due to the absence of target genetic locus) (Chen et al., 2018; Swarts and Jinek, 2019). Furthermore, other tools, including lateral-flow assays (LFA) and electronic readouts (Kaminski et al., 2021) as well as platforms, such as DETECTR (Chen et al., 2018) and SHERLOCK (Kellner et al., 2019) were adapted to visualize the results of CRISPR/Cas12a reaction and to check them with the naked eye. Together, they contribute to facilitating the application in point-of-care (POC) conditions and, thus, making it suitable to detect phytopathogens directly *in vivo*. These techniques also become useful and practical when integrated with isothermal amplification methods, such as loop-mediated isothermal amplification (LAMP) (Notomi et al., 2000) or recombinase polymerase amplification (RPA) (Piepenburg et al., 2006), enabling the amplification of target nucleic acid under field conditions at constant 30–40°C without a PCR machine (Kumar et al., 2018). Several isothermal amplification methods, including RPA, recombinase polymerase amplification Exo (RPA-EXO, using the probe as a target for the exonuclease, resulting in the separation of the fluorophore from the quencher and the subsequent release of a fluorescence signal), and RPA-lateral-flow test (LFT, a lateral-flow immunochromatographic assay based on the principle of colorimetric detection using gold nanoparticles), have been developed for pathogen detection (Ivanov et al., 2021; Jarvi et al., 2021). RPA provides rapid amplification at a constant temperature, while Exo RPA and RPA-LFT incorporate additional steps for enhanced sensitivity and readout simplicity for visual detection. These methods typically achieve results within 20–40 min, comparable to the proposed CRISPR/Cas12a-based assay (Kaminski et al., 2021). Furthermore, multiplex assays such as multiplex polymerase chain reaction (mPCR) and multiplex recombinase polymerase amplification (mRPA) coupled with the CRISPR-based assays enable simultaneous detection of multiple pathogens, enhancing diagnostic efficiency. These multiplex approaches are advantageous for comprehensive pathogen screening but can face challenges in maintaining sensitivity across numerous targets (Kaminski et al., 2021; Zeng et al., 2024; Zhou et al., 2024).

**Figure 1:**
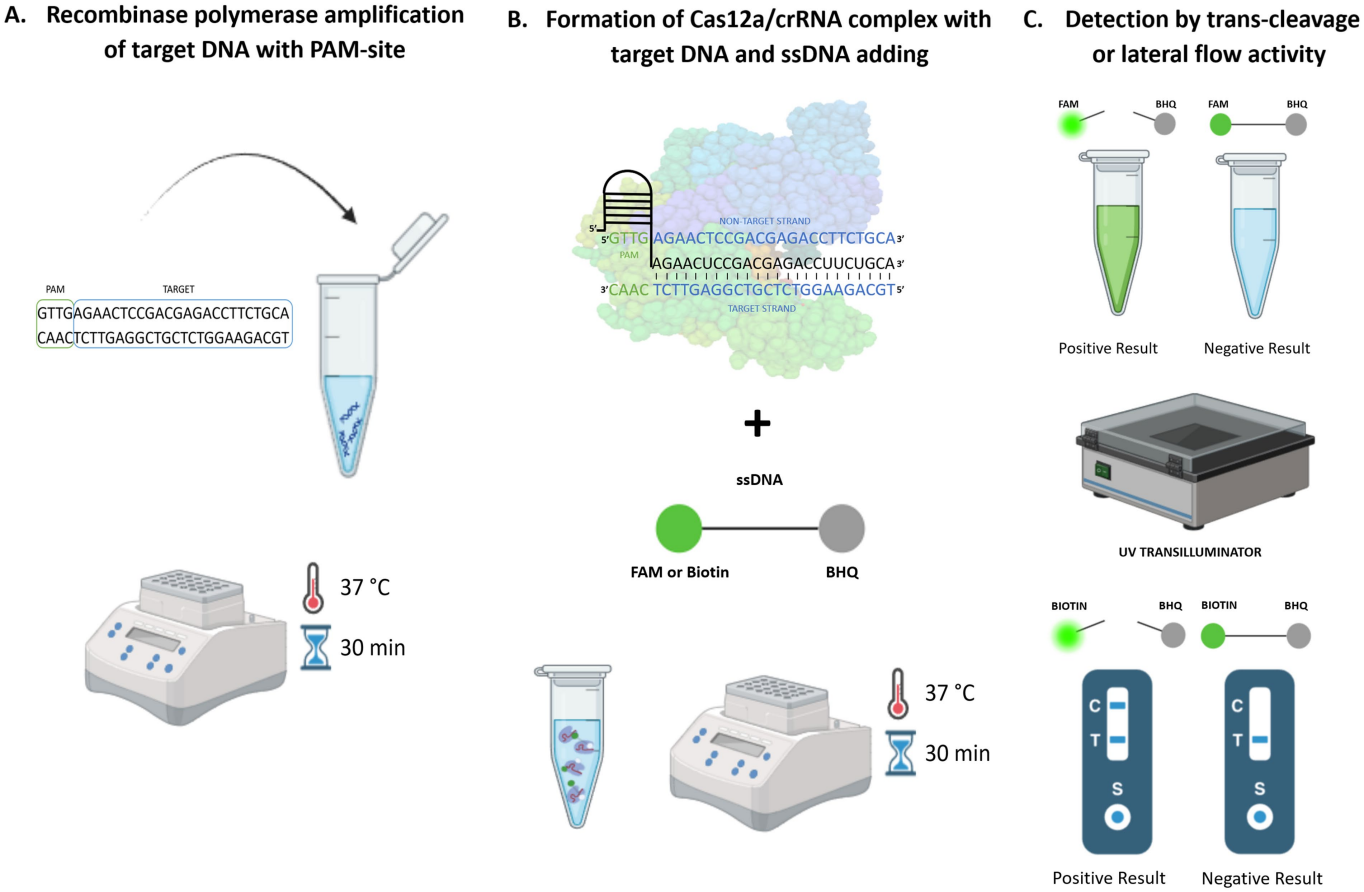
CRISPR/Cas12a-based coupled with RPA amplification method for pathogen detection. **(A)** Isothermal amplification, **(B)** CRISPR/Cas12a detection, **(C)** Visualization with the naked eye.

Current research confirms the sensitivity and specificity of these isothermal amplification methods even compared to conventional PCR (Yang et al., 2019; Jiao et al., 2021; Dong et al., 2024). Isothermal amplification of target nucleic acid directly in the field followed by the reaction with CRISPR/Cas12a and subsequent visualization of the results with the naked eye remarkably broadens the method’s potential to detect pathogenic DNA with high accuracy, sensitivity, and robustness (Qiu et al., 2022; Srivastava and Prasad, 2023). The mechanism of the CRISPR/Cas12a detection method coupled with isothermal amplification is presented visually in Figure 1.

Many reports have been published about the CRISPR/Cas12a detection method for a significant range of genetic targets. It was successfully applied to detect human bacterial (Luo et al., 2023) and viral pathogens (Yin et al., 2021), track antibiotic resistance (Rabaan et al., 2023), detect organophosphorus pesticides (Fu et al., 2022), and transgenic crops (Zhu et al., 2022). These reports confirm the remarkable potential of the CRISPR/Cas12a method for diagnostic purposes, including point-of-care (POC) assays. In agriculture, the CRISPR/Cas12a technology was applied to detect and identify several livestock pests and a diverse range of plant pathogens, including fungi, bacteria, viruses, and insects (Zhang, 2022; Tanny et al., 2023). For example, the CRISPR/Cas12 method was utilized to detect significant wheat pathogens, including *Fusarium asiaticum* and *Fusarium graminearum* as well as *Puccinia striiformis* f. sp. *tritici* and *Magnaporthe oryzae Triticum* (Kang et al., 2021; Mu et al., 2022; Sánchez et al., 2022; Zhang et al., 2023). Moreover, field applications of this method were also successfully demonstrated, such as for *Leptosphaeria maculans* in rapeseed (Lei et al., 2022) and *Verticillium dahliae* in sunflower, cotton, and potato (Wang et al., 2023), further suggesting its field-deployable implementation potential. However, as stated above, the CRISPR/Cas12a technology was not intensively studied for *Alternaria*, a serious global plant pathogenic fungal species. Among the *Alternaria* species reported to infect wheat grains, *Alternaria alternata*, *Alternaria infectoria*, *Alternaria tenuissima*, and *Alternaria triticina* were reported as the most prevalent species. *A. alternata*, in particular, frequently associated with black-point kernels diseases, a significant threat to wheat crops (Somma et al., 2019; Turzhanova et al., 2020). Detection and identification of *Alternaria* is considered a complex challenge. Morphology- and PCR-based assays as well as phylogenetic and metabolite profiling methods were deployed for *Alternaria* identification in different samples (Simmons, 2007; Lawrence et al., 2013; Woudenberg et al., 2013; Chakdar et al., 2019; Patriarca et al., 2019), including cereal samples (Zur et al., 2002). Regarding CRISPR/Cas12a methods, such methods were recently reported for citrus-associated *Alternaria* genes and the potato pathogen *Alternaria solani* (Liu et al., 2022; Guo H. et al., 2023; Ma et al., 2024). However, to the best of our knowledge, no information is available in the current literature on the detection of *Alternaria* spp. in wheat samples using the CRISPR/Cas12a technology. This presents a critical gap, as *Alternaria* spp. pose a significant risk to wheat quality and yield. Therefore, the present study aimed to address this gap by developing a CRISPR/Cas12a-based method with RPA amplification specifically for *Alternaria* detection in wheat grain samples.

## 2 Materials and methods

### 2.1 Fungal isolates, DNA extraction, and identification

#### 2.1.1 Isolation of fungi from cereal grain samples

Fungal strains were isolated from wheat and barley grain samples collected from different cultivars grown in Northern Kazakhstan, each sample containing 100 seeds. The seed surface was sterilized with 96% ethyl alcohol or ethanol (EtOH), followed by quick flaming. Sterilized seeds were placed in Petri dishes containing Potato Dextrose Agar (PDA: 49 g/L, HiMedia, pH was adjusted at 5.0–5.5 with 80% lactic acid), 25 grains per Petri dish, at 4 replicates. The grains were incubated in a thermostat at 25–27°C for 7–10 days. Following incubation, colonies resembling *Alternaria* morphology were selected based on a visual examination of fungal mycelium. Pure cultures were obtained by re-inoculating selected strains on PDA and subsequent cultivation at 26°C for 7–14 days. Initial optical microscopic identification was performed using a digital binomial microscope (Altami Bio 1, Saint Petersburg, Russia). Strains exhibiting visual characteristics typical of *Alternaria* were subjected to subsequent molecular identification.

#### 2.1.2 Fungal DNA extraction

This study used two methods of fungal DNA extraction from wheat samples. Initially, two strains attributed visually as *Alternaria* spp., as well as three strains with different morphologies, were chosen for the development of the method, including selection of target genetic locus, construction of positive control, optimization of isothermal amplification method, and evaluation of *cis* and *trans*-activities. Upon the method development, an additional six strains of *Alternaria* and 1 strain with different morphology were selected for method validation.

The commonly applied cetyltrimethylammonium bromide (CTAB) method described by Turzhanova et al. (2020) with modifications was employed for DNA extraction in the first phase, while commercial nucleic acid extraction kit (GF-1, Vivantis, Kuala Lumpur, Malaysia) was used to extract DNA in the second phase. All fungal DNA was extracted from mycelium grown in liquid Czapek medium (30 g/L sucrose, 2 g/L NaNO_3_, 1 g/L KH_2_PO_4_, 0.5 g/L MgSO_4_·7H_2_О, 0.5 g/L KCl, 0.01 g/L FeSO_4_·7H_2_О) for 7–10 days, depending on the strains. For the CTAB method, mycelium (50–100 mg) was collected in 2 mL Eppendorf tubes and frozen at −80°C for 1 h, followed by grinding into a powder. A 700-uL 1.5% CTAB (AppliChem, Darmstadt, Germany) solution (pH 6.7) was added to the ground mycelium, and the mixture was vigorously shaken and incubated at 55°C for 1 h. Proteinase K (Thermo Scientific, Germany) and 2-mercaptoethanol (Sigma-Aldrich, Darmstadt, Germany) were added to the CTAB solution prior to mixing with the mycelium at a final concentration of 0.2 mg/mL and 1%, respectively. Incubated samples were centrifuged at 13,000 g, 25°C for 15 min (Eppendorf 5425R, Hamburg, Germany), and 600-μL supernatant was mixed with an equal amount of 24:1 chloroform:isoamyl alcohol (Sigma-Aldrich, St. Louis, MO, United States of America) and intensively shaken for 5 min. Samples were centrifuged under the same conditions, and 500 μL of the obtained supernatant was mixed with 25-μL 3 M sodium acetate (AppliChem, Darmstadt, Germany) (pH 5.2) and 400-μL 2-propanol (AppliChem, Darmstadt, Germany). The mixture was shaken gently and centrifuged at 1,680 g, 25°C for 5 min. The supernatant was discarded, and the DNA pellet was washed twice with 1 mL 96% EtOH, followed by centrifugation at 9,700 g for 10 min. To remove the residual EtOH, the tubes were inverted on filter paper for 5 min and then incubated at 37°C for 1–2 min. DNA was eluted by adding 50-μL TE buffer (1-mM EDTA, 10-mM Tris-HCl, pH 8.0) and incubating at 37°C for 30 min. If a non-diluted pellet appeared, tubes were centrifuged at 600 g, 25°C for 2 min, and DNA-containing supernatant was transferred to a new tube.

For the nucleic acid extraction kit, DNA extraction was performed according to the manufacturer’s instructions, including treatment with proteinase K (20 μL from 20 mg/mL, provided by the manufacturer) and RNAse A (10 μL from 20 mg/mL, Thermo Scientific), and elution of DNA in MG-H_2_O.

The concentration (ng/μL) and purity (260/280 and 260/230 ratio) of the obtained DNA were measured by checking 0.5-μL DNA spectrophotometrically on NanoDrop One (Thermo Scientific, Madison, United States). DNA was stored at −20°C until use.

#### 2.1.3 Identification of the fungal isolates

The selected fungal strains were identified based on the partial sequence of the internal transcribed spacer (ITS) region using widely utilized ITS1/ITS4 primers (White et al., 1990) under the PCR parameters specified in Table 1.

**Table 1:** PCR primers and amplification conditions used in this study.

Primer	Primers (5′–3′)	PCR program	References
Identification assay			
ITS1	TCCGTAGGTGAACCTGCGG	98°C, 30 s (1 cycle) 98°C, 10 s; 55°C, 15 s; 72°C, 30 s (30 cycles) 72°C, 5 min (1 cycle) 4°C—storage	White et al. (1990)
ITS4	TCCTCCGCTTATTGATATGC		
*Alternaria* specificity assay			
Aalt-For	GTGCCTTCCCCCAAGGTCTCCG	94°C, 3 min (1 cycle) 94°C, 30 s; 72°C, 1 min (35 cycles)	Kordalewska et al. (2015)
Aalt-Rev	CGGAAACGAGGTGGTTCAGGTC		

PCR Master Mix for the ITS gene sequences contained (given at the final concentrations) 2-2 μL forward and reverse primers (0.4 μM), 5-μL deoxynucleotide triphosphate (dNTP) mix (0.2 mM), 10-μL HF buffer (1×), 1 μL Phusion Polymerase (Thermo Scientific), 1-μL DNA template, and the final volume at 50 μL was adjusted with diethylpyrocarbonate (DEPC)-treated H_2_O. PCR was performed in a T100 Thermal Cycler (Bio-Rad, Singapore). PCR products were loaded in 1% agarose gel stained with Ethidium Bromide at 0.1 mg/mL concentration, and checked using horizontal gel electrophoresis in 1× TAE buffer (Merck, Darmstadt, Germany). The electrophoresis was run at 90 V for 30–40 min, and the results were visualized using GelDoc Go Imaging System (Bio-Rad, USA). PCR amplicons (20 μL) were treated with 0.5 μL FastAp (1 U/μL) and 0.25 μL ExoI (20 U/μL) enzymes (Thermo Scientific) followed by incubation in a water bath at 37°C for 30 min and subsequent inactivation in a PCR machine at 85°C for 15 min. 3 μL of the samples obtained were mixed with 0.5-μL Big Dye (Thermo Scientific, Vilnius, Lithuania), 2-μL Big Dye sequencing buffer (Applied Biosystems, Warrington, UK), 1-μL corresponding primers (from 10-μM stock), and adjusted with DEPC-H_2_O up to 10 μL. The mixture was briefly vortexed, centrifuged, and subjected to the PCR sequencing program [96°C, 1 min (1 cycle), 96°C, 10 s; 55°C, 5 s; 60°C, 4 min (25 cycles), 10°C, 20 min (1 cycle)]. PCR amplicons were purified subsequently with EtOH-NaOAc mixture, 70% EtOH, and formamide and stored at −20°C. Prior to sequence analysis, samples were denatured in a PCR machine at 96°C for 5 min, loaded in a 96-well plate, and subjected to Sanger sequencing. The obtained sequences were proceeded to the National Center for Biotechnology Information (NCBI) Basic Local Alignment Search Tool (BLAST) analysis; the accession numbers are presented in Table 2.

**Table 2:** Fungal strains used in this study.

Fungal strain	Genus/species	NCBI deposited accession number
Method development and validation		
4/1	*Alternaria* sp.	PQ056909
7/2	*Alternaria* sp.	PQ056910
8/1	*Alternaria* sp.	PQ056911
8/5	*Alternaria* sp.	PQ056912
8/7	*Alternaria* sp.	PQ056913
11/1	*Alternaria* sp.	PQ056914
41/1	*Alternaria* sp.	PQ056915
42/1	*Alternaria* sp.	PQ056916
Sensitivity assay		
8/1 (gDNA)	*Alternaria* sp.	PQ056911
Specificity assay		
22/1	*Nigrospora oryzae*	PQ056917
465	*Bipolaris sorokiniana*	PQ056918
1/3	*Fusarium equiseti*	PQ056919
25/1	*Fusarium acuminatum* (*Fusarium tricinctum* species complex)	PQ056920

### 2.2 Selection and construction of positive control

The β-tubulin gene reported in the literature for PCR-based identification of *A. alternata* (Kordalewska et al., 2015) was selected as a potential target genetic locus for identifying *Alternaria* spp. Two initial strains of *Alternaria* spp. 8/1 and 8/5, as well as *B. sorokiniana* 465 and *Fusarium acuminatum* 25/1 isolates, were tested to validate this gene’s specificity using the PCR program and the corresponding primers listed in Table 1. The PCR MasterMix contained 1-1-μL 10-μM forward and reverse primers (0.4 μM each as the final concentration), 3-μL 25-mM MgCl_2_ (3 mM as the final concentration), 0.5-μL 10-mM dNTP (0.2-mM final concentration), 2.5-μL 10× Taq buffer (Thermo Scientific), 1-μL Taq polymerase (Thermo Scientific), 1-μL gDNA, and the final volume was made up to 25 μL with diethylpyrocarbonate (DEPC)-H_2_O. As described above, the PCR results were verified by horizontal gel electrophoresis, and strain 8/1 was selected to obtain positive control.

According to the commercial protocol, the PCR amplicon of 8/1 was cloned into the pJET cloning kit (Thermo Scientific). The obtained positive control was the target genetic locus sequencing using the sample treatment described in the molecular identification section. Multiple alignments of the obtained β-tubulin of *Alternaria* species, as well as other wheat pathogens (Supplementary Figure S9), were performed in VectorNTI to support the feasibility of this selected molecular marker.

### 2.3 Design of the RPA primers and crRNA

The obtained positive control sequence was applied to design the relevant crRNA and primers required to proceed RPA reaction using VectorNTI software (Lu and Moriyama, 2004). The following parameters, such as melting temperature of 40–65°C, GC content of 40–70%, and length of 30–35 bp, were set for the RPA primers (both forward and reverse) using the criteria suggested in the TwistAmp kit manual. A corresponding 24 bp-long crRNA was designed based on the protospacer adjacent motif (PAM) with its target as GTTG found in the sequence between the designed RPA primers.

crRNA was synthesized using the HiScribe T7 Quick High Yield RNA Synthesis Kit (New England Biolabs, Ipswich, MA, United States of America) according to the instructions provided by the manufacturer. The obtained RNA was purified with the Monarch RNA Cleanup Kit (New England Biolabs, Ipswich, MA, United States of America), following the commercial protocol, and its amount and quality were analyzed spectrophotometrically using NanoDrop, as described previously. The main stock of crRNA was stored at −80°C and utilized only to prepare routinely used laboratory stock at 10 μM, which was kept at −20°C.

### 2.4 *Cis-*activity examination of MbCas12a

The positive control plasmid, containing the cloned target sequence of β-tubulin served as the genetic target, was amplified using PCR with RPA-designed primers. The PCR MasterMix contained the following final concentrations: 0.4-μM forward and reverse primers, 0.2 mM dNTP mix, 1× HF buffer, 1-μL Phusion DNA polymerase, and 0.1-μL plasmid DNA (119 ng/μL), with DEPC-H_2_O added to a final volume of 25 μL. PCR was performed with the following conditions: initial denaturation at 98°C for 30 s, followed by 30 cycles of 98°C for 10 s, 63°C for 15 s, and 72°C for 20 s, with a final annealing step at 72°C for 5 min. PCR products were analyzed using the horizontal gel electrophoresis method and purified with the QIAquick PCR Purification kit (QIAQEN GmbH, Hilden, Germany) according to the manufacturer’s instructions. Purified PCR amplicons were eluted in 50-μL DEPC-H_2_O, quantified using a NanoDrop spectrophotometer, as depicted above, and stored at −20°C until use.

The recombinant MbCas12a protein obtained from *Moraxella bovis* in our laboratory, as described previously (Shaizadinova et al., 2023), was employed in all corresponding studies. The obtained protein was verified by reverse-phase C18 liquid chromatography-tandem mass spectrometry (LG-MS/MS), and laboratory stock was stored in 50% glycerol at −20°C. The *cis*-activity of MbCas12a according to the designed crRNA was verified by incubating MbCas12a, crRNA, and the purified PCR amplicon followed by subsequent horizontal gel electrophoresis and visualization using a GelDoc System. The MasterMix for the reaction was prepared to a total volume of 20 μL and included (at final concentrations) 1× NEB 2.1 buffer (New England Biolabs, Ipswich, MA, United States of America), 1-μM MbCas12a, 2-μM crRNA, and 10-μL DEPC-H_2_O. In variants where MbCas12a, crRNA, or PCR products were omitted, the volume of DEPC water was adjusted, accordingly. For variants testing the effect of different concentrations of MbCas12a, the amount of crRNA was kept at 2× of MbCas12a, with the remaining volume adjusted with DEPC-H_2_O. The mixture was initially incubated at 25°C for 15 min (to allow the formation of a ribonucleoprotein complex between crRNA and MbCas12a), followed by the addition of 1 μL purified PCR product, and subsequent incubation at 37°C for 30 min. The reaction was terminated upon incubation by adding 2-μL proteinase K and incubating at 37°C for 10 min. The entire reaction volume was loaded in 2% agarose gel and run at 90 V for 30–40 min. To validate the results precisely, two commercial markers (SM0371 and SM0333, Thermo Scientific, Vilnius, Lithuania) were applied in *cis*-activity assays to validate the results precisely. The *cis*-activity was examined visually by verifying the obtained bands with expected sizes, resulting from the cleavage of the amplified product by MbCas12a guided by the designed crRNA.

### 2.5 Method development and validation

#### 2.5.1 RPA reaction

Selected genetic locus and designed RPA primers were tested in RPA reaction using the TwistAmp Basic kit (TwistDX, Cambridge, United Kingdom). The RPA MasterMix included 2.4 μL of forward and reverse primers each (10-μM stock), 29.5-μL rehydration buffer, and 13.2-μL DEPC water. The mixture was briefly mixed, added to a TwistAmp tube containing lyophilized substrates, and mixed with a pipette 2–3 times. Subsequently, 2.5-μL 280-mM magnesium acetate was added to the mixture followed by the addition of 1 μL of the DNA sample. The exact amount of DEPC-H_2_O was added to negative control samples. The reaction was carried out in a thermal block (Eppendorf Thermotat C, Hamburg, Germany) for 20 min at 39°C, and the obtained RPA products were employed in further analyses, including verification via gel electrophoresis, GelDoc documentation, fluorescence assays, as well as real-time read-out test, specificity, and sensitivity analysis.

#### 2.5.2 *Trans*-activity evaluation and fluorescence readout

The collateral or *trans*-activity of MbCas12a toward the ssDNA reporter was verified using a reaction mixture containing MbCas12a, crRNA, and ssDNA (FAM-reporter-5-BHQ-1, TTATT). The reaction mix comprised 1× NEB 2.1 buffer (New England Biolabs, Ipswich, MA, United States of America), 100-nM MbCas12a, 100-nM crRNA, 5-μM ssDNA reporter, and 3-μL RPA product, given at the final concentrations. The final volume was made up of 30 μL with DEPC-H_2_O. The initial mixture of MbCas12a, crRNA, NEB buffer, and DEPC-H_2_O was incubated at 25°C for 15 min to allow MbCas12a and crRNA to assemble into a complex. After this preliminary incubation, 1-μL ssDNA and 3-μL RPA amplicons were added to the mixture and incubated at 37°C for 180 min. In addition to the negative control from the RPA reaction, DEPC-H_2_O added instead of RPA products served as the additional negative control to track any contamination during protocol employment. *Trans*-activity of MbCas12a was verified by a visual examination of the fluorescence signal resulting from the cleavage of the ssDNA reporter by MbCas12a. This cleavage occurs after the conformational change of Cas12a when it, guided by crRNA, detects and cuts the target sequence in the RPA amplicons. The samples were checked at 10, 20, 30, 60, 120, and 180 min under UV-illuminator (Vilber, Collégien, France) and photos were taken with a smartphone camera. The described method with the same fluorescence molecule (FAM-reporter-5-BHQ-1, TTATT) was applied for all further studies that required fluorescence detection with the naked eye. At the same time, DEPC-H_2_O was employed as a negative control in such assays.

#### 2.5.3 Sensitivity assays

Sensitivity assays were carried out to determine the detection limit of the proposed method using genomic DNA (gDNA) from strain 8/1. Based on the genome size of *Alternaria* as 32.30 Mb (Huang et al., 2021), the extracted genomic DNA was diluted at a 10-fold ratio to 42.2 × 10^−6^ ng/μL (ranging from 1,212,298 to 1.2 copies/μL). The diluted DNA samples were amplified using the RPA method, and the results were analyzed through gel electrophoresis and fluorescence assays. For fluorescence emission, the same FAM-labeled ssDNA reporter probe (FAM-reporter-5-BHQ-1, TTATT) utilized in the *trans*-activity evaluation assay above was applied. DEPC-H_2_O was applied as the negative control. Additionally, all samples underwent real-time (RT) fluorescence analysis to confirm the results obtained from the gel and fluorescence studies. The RT-readout assays followed the same protocol described for the *trans*-activity method, except that the samples were incubated in an RT-PCR C1000 Touch Thermal Cycler (Bio-Rad, Singapore). The RT-fluorescence program consisted of 30 cycles, with each cycle including a 50-s run followed by a 10-s measurement. The RT-fluorescence readout test was performed in three technical replicates.

#### 2.5.4 Method validation and its specificity

Six newly extracted DNA of *Alternaria* fungal strains (4/1, 7/2, 8/7, 11/1, 41/1, and 42/1) along with 2 *Alternaria* isolates (8/1 and 8/5) used in the previous assays were employed to validate the CRISPR/Cas12a detection method. DNA from all isolates was subjected to the RPA reaction. Acquired RPA amplicons were further analyzed using conventional gel electrophoresis and fluorescence assay to visualize the results and confirm the potential of the proposed method.

DNA from different fungal strains also isolated from wheat and barley grain samples were selected for specificity testing. These strains included *B. sorokiniana* 465, *F. acuminatum* (*Fusarium tricinctum* species complex) 25/1, *Fusarium equiseti* 1/3, and *N. oryzae* 22/1. All DNA samples were amplified by the RPA reaction. The obtained amplicons were analyzed by gel electrophoresis method and fluorescence assays to verify the specificity of the CRISPR/Cas12a-based identification method for *Alternaria* strains only.

All utilized protocols were carried out as described in the corresponding preceding sections.

### 2.6 Statistics, software, and reagents

All reagents and kits applied, unless specified, were purchased from Sigma-Aldrich and Thermo Scientific, respectively. All reagents applied, unless specified, were of molecular grade purity. The line chart for the RT-fluorescence signal was created in R (version 4.3.1; https://www.r-project.org/) and RStudio^1^ software using the *ggplot2* package (Wickham, 2016).

## 3 Results

### 3.1 Fungal isolates

**Figure 2:**
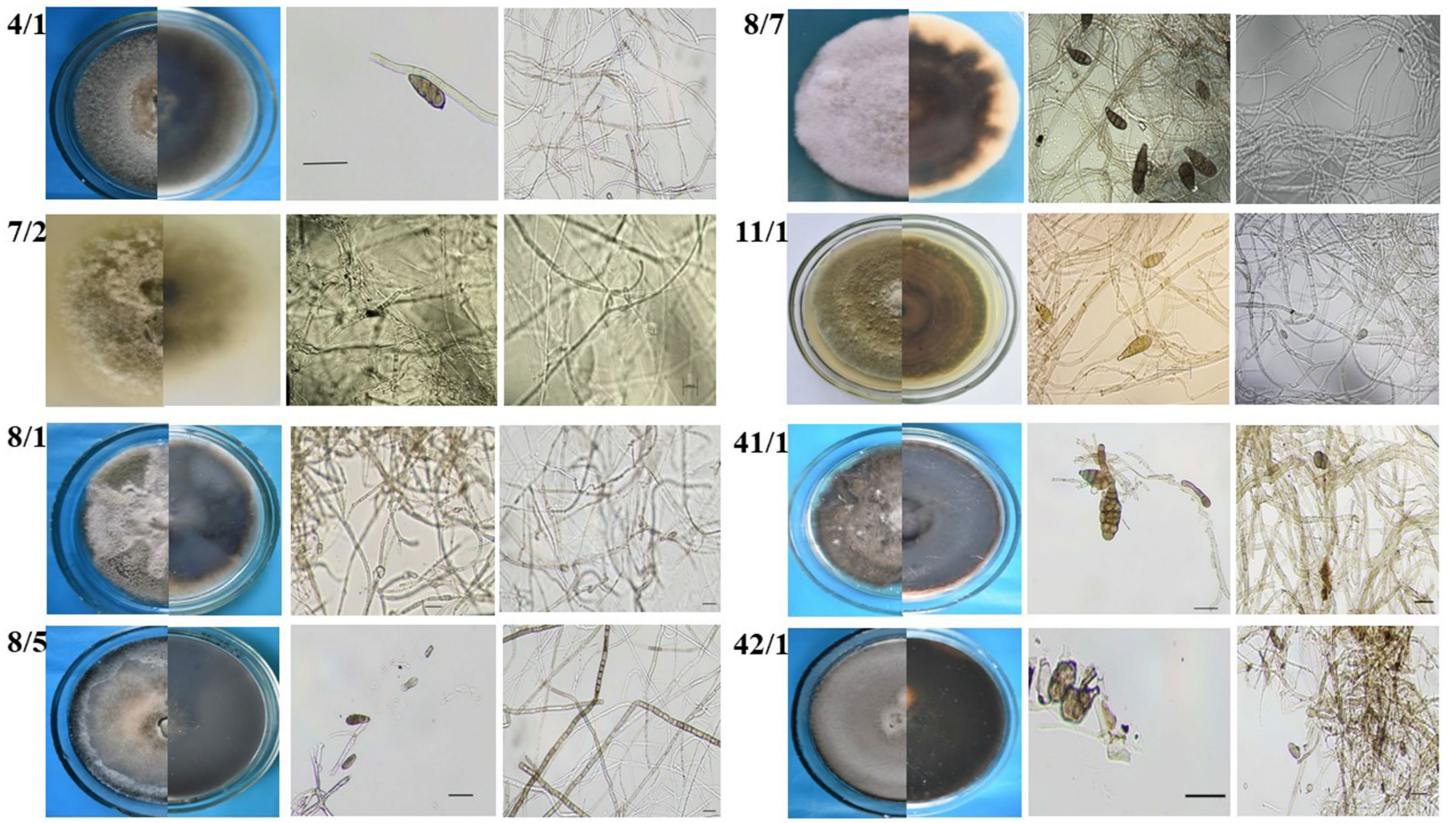
Preliminary morphology-based identification of *Alternaria* fungi isolated from wheat grain samples.

Twelve fungal strains possessing different morphology were isolated from wheat and barley grain samples collected in Northern Kazakhstan. Preliminary identification involved visual examination of fungal morphology and microscopic identification of the isolated strains. The morphology of *Alternaria* is typically characterized as hyphomycetes, exhibiting darkly pigmented multicellular conidia (Lawrence et al., 2016). However, *Alternaria* species exhibit different patterns depending on their morphological groups classified by Simmons and Roberts (1993). Eight strains exhibiting such typical *Alternaria* morphology (Figure 2) were selected for further assays.

Morphological characterization of the *Alternaria* mycelium and spores of the selected isolates was evaluated based on the cell morphology and structure, the presence or absence of spore chains and their type, and the size and type of conidia (Simmons, 2007). The morphological evaluation revealed that the isolates represent different *Alternaria* species, while subsequent analysis of their ITS region attributed all of them to *Alternaria* species (Table 2).

The remaining 4 strains possessing different morphology were identified based on the ITS region as *B. sorokiniana*, *F. acuminatum* [representing *F. tricinctum* species complex, Laraba et al. (2022)], *F. equiseti*, and *N. oryzae*. Among these isolates, three isolates, namely, *F. acuminatum* 25/1, *F. equiseti* 1/3, and *N. oryzae* 22/1, were isolated from wheat, while a single-strain *B. sorokiniana* 465 originated from barley grain samples. These species are frequently isolated from cereal samples worldwide, including Kazakhstan (Özer et al., 2020; Bozoğlu et al., 2022; Laraba et al., 2022). These strains were included in the assays to evaluate the specificity of the CRISPR/Cas12a method toward *Alternaria* strains.

### 3.2 Positive control and design of the RPA primers and crRNA

A purified plasmid containing the target locus of the β-tubulin gene obtained from cloned *Escherichia coli* DH5α cells served as the positive control for method development. The cloned target was sequenced and the sequence was subjected to VectorNTI analyses to design RPA primers and the corresponding crRNA.

Based on the obtained sequence one set of primers was generated according to the given parameters for primers described above. The forward (RPA-alt-alt-J-FW) and reverse primers (RPA-alt-alt-J-RV) were CCTTCCCCCAAGGTCTCCGACACCGTTGTC and AACGAGGTGGTTCAGGTCGCCGTAGGAGGG, respectively. Subsequently, the PAM site (GTTG) necessary for crRNA recognition of the target was found in the sequence between the listed primers and 24-bp long crRNA (MbCas12a-crRNA-Phyto-Alt-alt-J) was obtained as follows: TGCAGAAGGTCTCGTCTGAGTTCTatctacaaacagtagaaattccctatagtgagtcgtattagaatt.

### 3.3 *Cis*-activity evaluation

**Figure 3:**
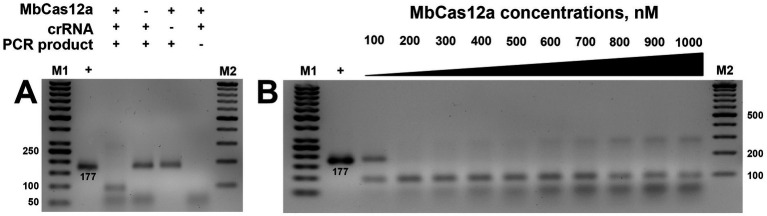
*Cis*-activity of MbCas12a **(A)** and the effect of its different concentrations **(B)**. The + and − signs in front of the reaction components are their presence and absence in the reaction, respectively. The + sign in the first sample is a PCR product added to the gel without any reaction. M1 and M2 are SM0371 and SM033 markers (Thermo Fisher Scientific, Vilnius, Lithuania), respectively. The incubation time of the reaction was 30 min in both assays.

The *cis*-activity of Cas12a is characterized by the ability of this type of CRISPR protein to cut the target dsDNA, resulting in sticky ends. This programmable on-target cleavage occurs under the guidelines of the corresponding crRNA-targeted T-rich PAM sequence (Li et al., 2018a). In our studies, we investigated the *cis*-activity of MbCas12a toward the target genetic locus of the β-tubulin gene, containing the total amplified region of 177 bp. The formation of the ribonucleoprotein complex of the designed crRNA and MbCas12a cleaved its target, resulting in two bands containing 93 and 84 bp, respectively (Figure 3A).

No cleavage was observed in variants where crRNA, MbCas12, or the genetic target (PCR product) was omitted in the reaction (Figure 3A). Furthermore, *cis*-activity of MbCas12a was observed starting at 200 nm, and further increase of *cis*-activity exhibited a concentration-dependent manner (Figure 3B). Only partial cleavage could be observed in the variant of MbCas12a of 100 nM.

**Figure 4:**
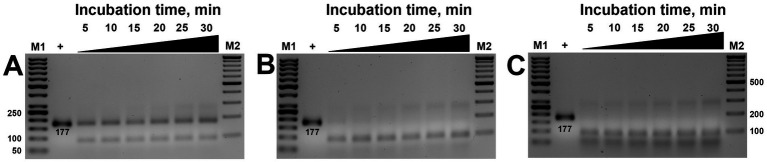
The effect of incubation time (5–30 min) on the *cis*-activity of MbCas12a (**A–C** are 100-, 200-, and 750-nM MbCas12a, respectively). The “+” sign in the first sample is PCR product served as positive control added in the gel without any reaction. M1 and M2 are the SM0371 and SM033 markers (Thermo Fisher Scientific), respectively.

Subsequent studies aimed to verify the necessary time required for the ribonucleoprotein complex to detect and cut the target. Time-lapse studies ranging from 5 to 30 min incubation revealed that visible cleavage occurred after 5 min. In contrast, complete substrate cleavage was observed in 10 min (Figure 4), except at 100 nM concentration, where only partial cleavage was observed (Figure 4A). These results were consistent regardless of whether low (200 nM) or high (750 nm) concentrations of MbCas12a were used (Figures 4B,C).

The *cis*-activity assay of MbCas12a toward the 177-bp long amplified target of the β-tubulin gene required minimal necessary concentrations and incubation time of 200 nM and 10 min, respectively.

### 3.4 Assessment of the method sensitivity

RPA amplicons of gDNA of *Alternaria* 8/1 strain were tested to evaluate the proposed method’s detection limit. The results were examined using gel electrophoresis followed by fluorescence studies. Additionally, the RT-fluorescence readout method was applied to validate the obtained findings precisely.

**Figure 5:**
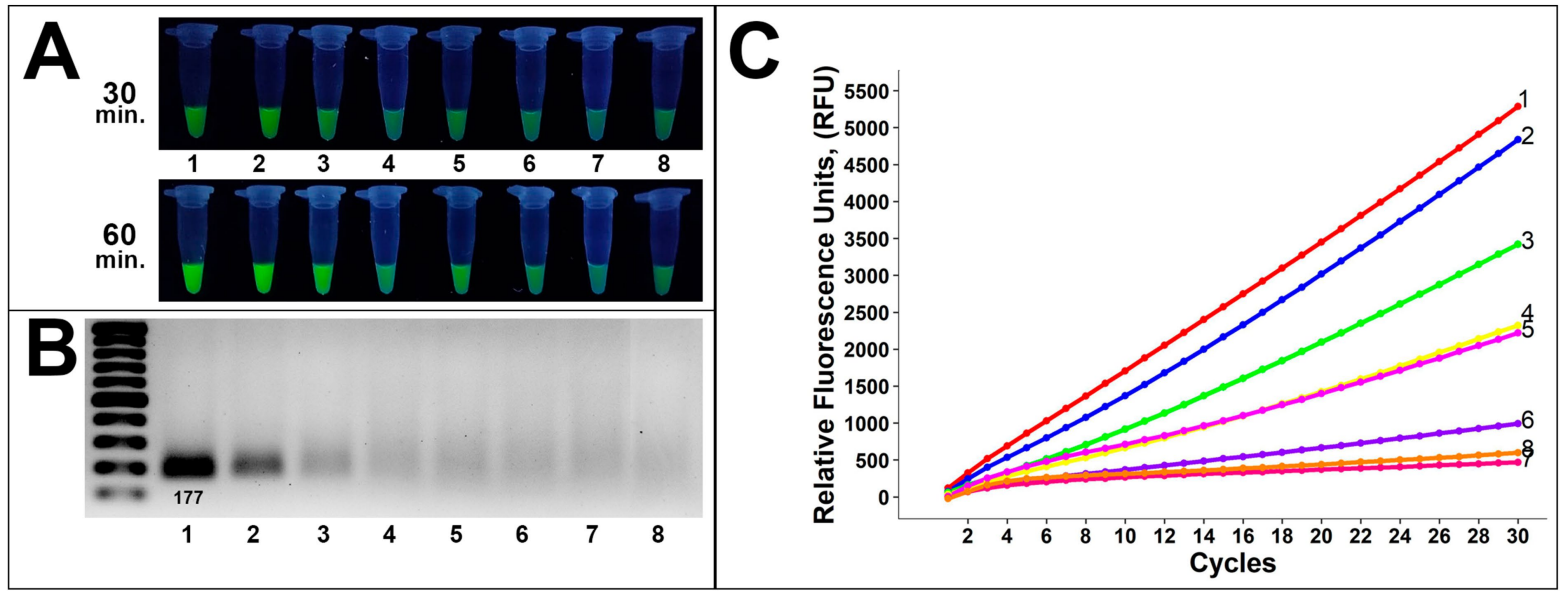
Evaluation of the method sensitivity toward gDNA of 8/1 *Alternaria* strain **(A)** fluorescence assays for potential on-site applications (incubation time was 30 and 60 min), **(B)** gel electrophoresis for the conventional method, **(C)** RT-fluorescence readout assays for validation. Samples 1–8 are 0, 10^−1^, 10^−2^, 10^−3^, 10^−4^, 10^−5^, 10^−6^, and water (negative control), respectively.

Analysis of gDNA demonstrated a sufficient sensitivity level on the gel analysis (Figure 5). Visual bands were observed up to 10^−4^ in gel electrophoresis and 10^−5^ dilution in the fluorescence assay, corresponding to approximately 1,212 and 121 copies, respectively (Figures 5A,B). Presumably, due to the lower DNA amount, the fluorescence signal after 60-min incubation was more sufficient compared to 30-min incubation, applied in all further assays below. These results were confirmed by subsequent RT-fluorescence assays in which the same samples demonstrated sufficient fluorescence unit quantities up to 121 copies (Figure 5C).

These findings showed a competent sensitivity level of the proposed method as earlier detection of crop pathogens is essential for manageable disease treatment. A limit of detection down to 100 copies is preassembly useful to verify the presence of *Alternaria* isolates before the visual symptoms appear, which is beneficial for sample evaluation *in vivo*.

### 3.5 Validation of the CRISPR-Cas12a method and its specificity

**Figure 6:**
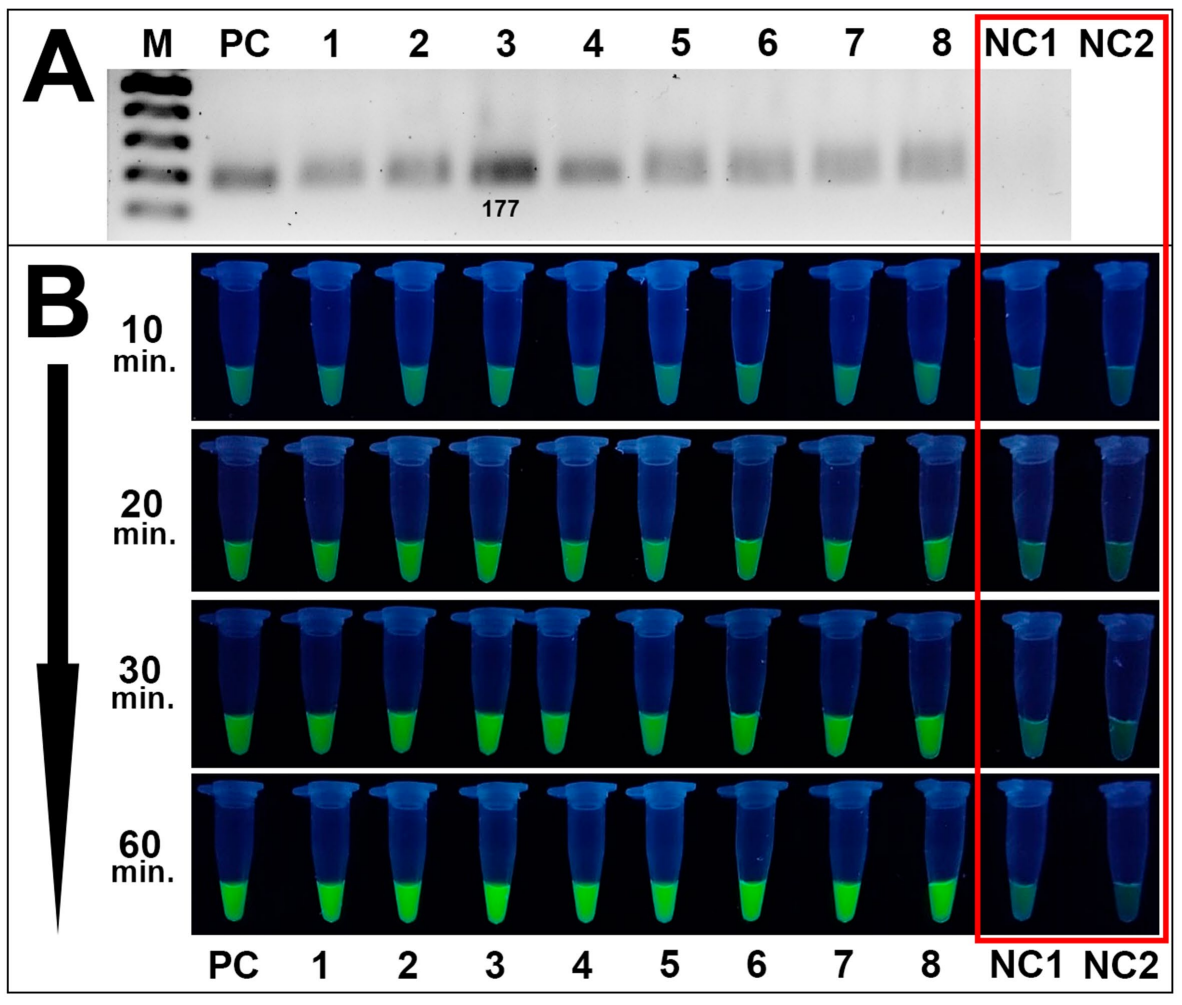
Validation of the CRISPR/Cas12a method toward *Alternaria* strains isolated from wheat grain samples. **(A)** RPA amplification results of 8 *Alternaria* isolates verified by the conventional gel electrophoresis method: PC is positive control (plasmid); samples 1–8 are *Alternaria* strains 4/1, 7/2, 8/1, 8/5, 8/7, 11/1, 41/1, 42/1, respectively; NC1 is negative control (DEPC-H_2_O of RPA amplification). M is an SM033 marker (Thermo Fisher Scientific). **(B)** Fluorescence assay for potential on-site detection: sample order is the same as listed in **A**; sample NC2 is additional negative control (DEPC-H_2_O of fluorescence assay). Both negative controls are highlighted in the red square. Incubation time was 10, 20, 30, and 60 min.

Method validation assays included the RPA reaction of DNA samples of 8 *Alternaria* isolates, visualization of the RPA products by gel electrophoresis, fluorescence assays, and specificity analysis toward *Alternaria* spp. The RPA method successfully amplified the target site of the β-tubulin gene in all tested *Alternaria* isolates (Figure 6A).

RPA products were further subjected to fluorescence assays utilizing the *trans*-activity of MbCas12a and the fluorescence emission of the ssDNA fluorescence molecule (FAM-reporter-5-BHQ-1, TTATT). In positive samples, where the target DNA is present, crRNA guides MbCas12a to the PAM site. Upon cleavage of the target DNA, MbCas12a undergoes a conformational change, activating its non-specific cleavage activity. Consequently, fluorescence-labeled ssDNA in the reaction is also cleaved by MbCas12a, releasing fluorescence molecules from the quenchers and generating a fluorescence signal. In negative samples, where no target DNA is present, no conformational change of MbCas12a occurs; consequently, ssDNA remains intact, and no fluorescence signal is detected. Analysis of *Alternaria* spp. showed efficient validation results for detecting *Alternaria* spp. by the CRISPR/Cas12a method, while no fluorescence signal was detected for negative control samples (Figure 6B).

**Figure 7:**
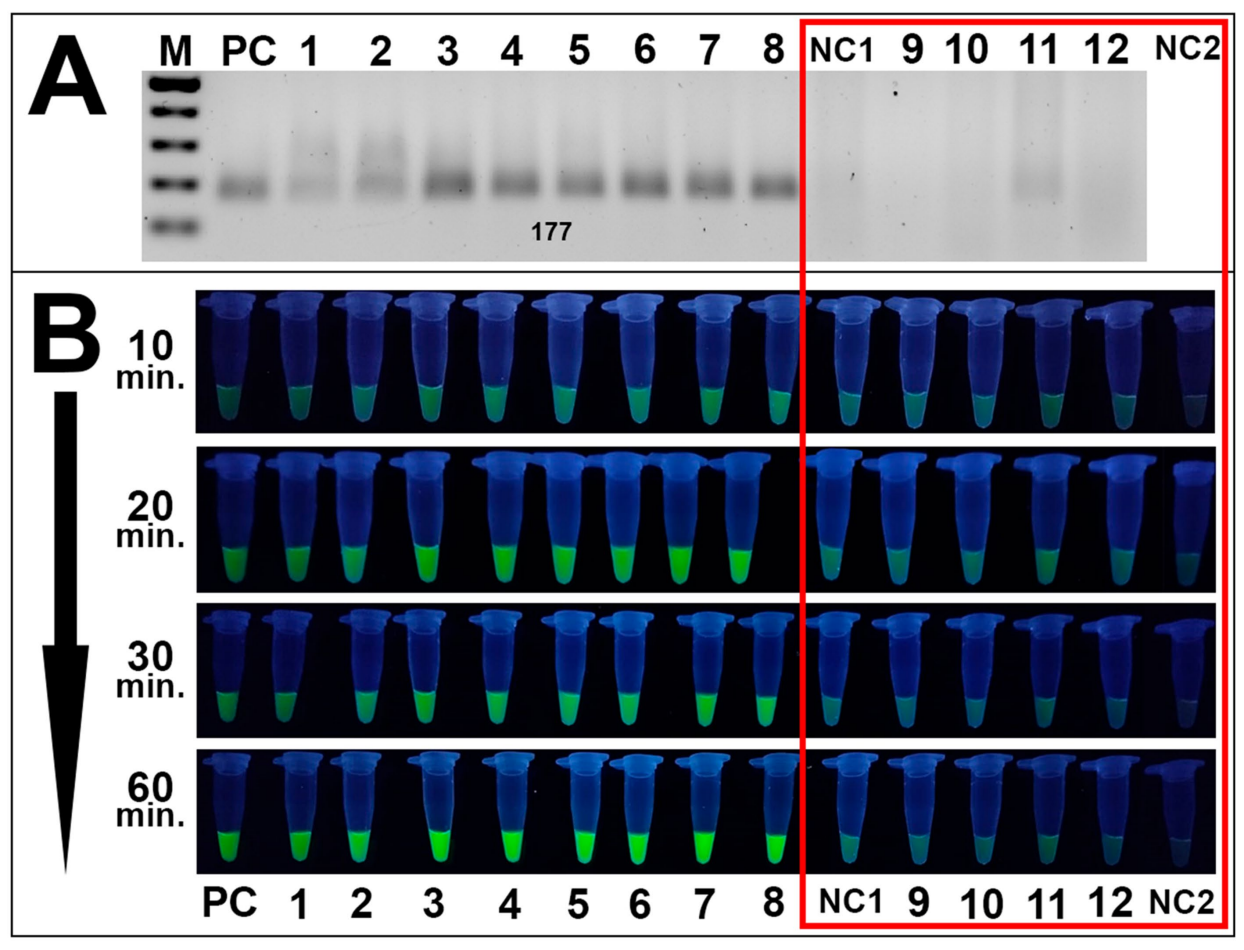
Specificity results of the CRISPR/Cas12a method. **(A)** Gel electrophoresis of the RPA amplification of fungal strains isolated from grain: PC is positive control (plasmid); samples 1–8 are *Alternaria* strains 4/1, 7/2, 8/1, 8/5, 8/7, 11/1, 41/1, and 42/1, respectively; NC1 is negative control (DEPC-H_2_O of RPA amplification); samples 9–12 are *B. sorokiniana* 465, *Nigrospora oryzae* 22/1, *F. equiseti* 1/3, and *Fusarium acuminatum* 25/1. **(A,B)** Visualization via fluorescence assays: sample order is the same as listed in **A**; sample NC2 is an additional negative control (DEPC-H_2_O of fluorescence assay). Both negative controls and non-*Alternaria* species are highlighted in the red square. Incubation time was 10, 20, 30, and 60 min.

Specificity assays also revealed high specificity in RPA analyses and subsequent fluorescence detection. Only *Alternaria* isolates were amplified during RPA assays while no amplicons were observed for other species during the specificity assays (Figure 7A). These results were validated via fluorescence testing: only *Alternaria* strains exposed visible fluorescence signal while the remaining fungal species isolated from grain samples exhibited no distinguishable from negative control samples signal (Figure 7B).

Both assays showed efficient results, suggesting the potential of the proposed method to detect *Alternaria* spp. using a UV-illuminator in the laboratory or a portable UV-lamp under field conditions Sufficient distinguishable from negative control samples fluorescence signal resulted as the cleavage of ssDNA reporter by MbCas12 after its recognition and cutting of the target β-tubulin was observed after 20–30 min of incubation. No clear fluorescence signal was exhibited by either H_2_O underwent during the RPA reaction as a negative control, or the water sample added as an additional negative control during fluorescence assays. Furthermore, none of the tested fungal species isolated from grain samples displayed a fluorescence signal, suggesting the method’s specificity to *Alternaria* spp. This high sensitivity is underscored by the dual-specificity of the technique, including a selection of the selective RPA primers and recognition of chosen PAM sites by designed crRNA.

## 4 Discussion

“Pest” usually includes more than 67,000 species of plant pathogenic microorganisms, weeds, and animals that negatively affect crop production (Oerke et al., 1994; Chandler et al., 2011). Up-and-coming as well as indigenous pathogens represent a serious obstacle in crop protection management globally. Their precise, fast, and robust detection, identification, and quantification are key to successfully applying corresponding defense strategies (Miller et al., 2009; Francesconi, 2022). To date, various detection methods are available, with the choice depending on factors such as the intended purpose, service requirements, equipment availability, and budget. Supplementary Table S1 presents a comparative overview of different diagnostic assays, illustrating the CRISPR/Cas12a-RPA assay’s advantages in speed and specificity relative to conventional detection methods. For example, the RPA-CRISPR/Cas12a-based method for citrus-associated *Alternaria* genes costs approximately 5.4 USD. It requires 2.5 h compared to 21.1 USD and 3.5 h of the PCR-based detection method (Liu et al., 2023).

CRISPR/Cas12a-based technology coupled with isothermal amplification is currently one of the most promising methods due to its detection at the molecular level, accuracy, robustness, and sensitivity. So far, research carried out with this method covers all crucial areas, such as clinical microbiology, agriculture, and food safety, including even the detection of organophosphorus pesticides and mycotoxins (Fu et al., 2022; Chen S. et al., 2024). Currently, various protocols are available to detect human viral and bacterial pathogens and livestock pests. In crop protection management, numerous studies have also reported the successful implementation of the CRISPR/Cas12a method in detecting various pathogenic species in different plant species, including model plants, trees, cereals, fruits, and vegetables (Table 3).

**Table 3:** Application potential of CRISPR/Cas12a-based method to detect crop pathogens.

Plant pathogenic species	Plant infected	Target genetic locus	Amplification method	References
Fungal pathogens				
*Verticillium dahliae*	*Cotinus coggygria* (smoke trees)	Translation elongation factor 1-alpha (*tef1α*)	RPA	Chen Q. et al. (2024)
	*Gossypium herbaceum* (cotton)	178-bp sequence	LAMP	Fang et al. (2024)
	*Helianthus annuus* (sunflower), *G. herbaceum* (cotton), *Solanum tuberosum* (potato)	*GAPDH* gene	RPA	Wang et al. (2023)
*Diaporthe aspalathi*, *Diaporthe caulivora*	*Glycine max* (soybean)	Specific single-copy gene	LAMP, RPA	Dong et al. (2024)
		Transcription elongation factor 1-alpha (*tef1*)	RPA	Sun et al. (2024)
*Fusarium circinatum*	*Cedrus deodara*	*Fcir2067* gene	RPA	Chen et al. (2023)
*Alternaria solani*	*S. tuberosum* (potato)	5.8S rRNA gene	LAMP	Guo H. et al. (2023)
*Phytophthora sojae*	*G. max* (soybean)	*Ypt1* gene	RPA	Guo et al. (2023a)
*Phytophthora ramorum*	*Rhododendron* × *pulchrum*	*Pr52094* gene	RPA	Guo et al. (2023b)
*Fusarium asiaticum*	*Triticum aestivum* (wheat), *Zea mays* (maize)	*CYP51C*	RPA	Zhang et al. (2023)
*Leptosphaeria maculans*	*Brassica napus* (rapeseed)	ITS	RPA	Lei et al. (2022)
*Alternaria*	Citrus, tomato, and apple	18S ribosomal RNA gene, ITS	PCR, RPA, rolling circle-amplification, RCA	Liu et al. (2022), Liu et al. (2023), and Ma et al. (2024)
*Fusarium graminearum*	*T. aestivum* (wheat)	ITS, transcription elongation factor 1α (EF1α)	PCR	Mu et al. (2022)
*Puccinia striiformis* f. sp. *tritici*, *Magnaporthe oryzae Triticum*	*T. aestivum* (wheat)	ITS, β-tubulin, MoT 6099 and MoT 6098 sequences	RPA	Sánchez et al. (2022)
*M. oryzae Triticum*	*T. aestivum* (wheat)	*MoT-6098* and *MoT-6099* genes	LAMP, RPA	Kang et al. (2021)
*Elsinoë fawcettii*	Citrus	Internal transcribed spacers (IST) 1 and 2	RPA	Shin et al. (2021)
*Magnaporthe oryzae*	*Oryza sativa* (rice)	*SDH1*, *TEF1*	RPA	Zhang et al. (2020)
Plant viruses				
*Maize chlorotic mottle virus*	*Zea mays* (maize)	Coat protein	Reverse transcription-recombinase-aided amplification	Duan et al. (2022)
*Tobacco mosaic virus*, *Tobacco etch virus*, *Potato virus X*	*Arabidopsis thaliana*, *Nicotiana benthamiana* (benth)	Coat protein	RT-PCR, Reverse Transcription-RPA (RT-RPA)	Marqués et al. (2022)
*Tomato mosaic virus*, *Tomato brown rugose fruit virus*	*Solanum lycopersicum* (tomato)	ORF1 region	RT-PCR	Alon et al. (2021)
*Apple necrotic mosaic virus*, *Apple stem pitting virus*, *Apple stem grooving virus*, *Apple chlorotic leaf spot virus*, *Apple scar skin viroid*	*Malus* × *domestica* (apple)	40-nt target dsDNA	RT-RPA	Jiao et al. (2021)
*Tomato yellow leaf curl virus*, *tomato leaf curl New Delhi virus*	*S. lycopersicum* (tomato)	coat protein	LAMP	Mahas et al. (2021)
*Beet necrotic yellow vein virus*	*Beta vulgaris* (sugarbeet)	BNYVV RNA-1	RT-RPA	Ramachandran et al. (2021)
*Potexvirus*, *Potyvirus*, *Tobamovirus*	*N. benthamiana* (benth)	Coat protein	RT-RPA	Aman et al. (2020)
Bacterial pathogens				
*Dickeya solani*	*S. tuberosum* (potato)	SOL-C region of unannotated gene	RPA	Kurbatov et al. (2024)
*Candidatus* Phytoplasma trifolii	*S. tuberosum* (potato)	16S-23S rDNA ITS regions	RPA	Wheatley et al. (2022)
*Xanthomonas arboricola* pv. *pruni*	*Prunus persica* (peach)	*ftsX* gene	RPA	Luo et al. (2021)

The wide cover of different pathogens detectable by the CRISPR/Cas12a method developed in the past 5 years listed in Table 3 confirms the application potential of this technology in agriculture. This method is very important to detect pathogens before visual symptoms, facilitate the selection of related protection measures, prevent outbreaks, monitor fields, and sustain stable crop production. This nucleic acid-based method can be applied in the POC applications to detect phytopathogens, including dangerous pests, such as *Alternaria* species, directly under field conditions. Altogether, it will facilitate providing food security—mitigating one of the most important global issues—as stated above.

In this study, we developed the CRISPR/Cas12a method coupled with RPA amplification to detect *Alternaria* spp. isolated from wheat grain samples. This method implementing both RPA as the most prevalent isothermal amplification method (Table 3) and the advanced CRISPR/Cas12a-based method which was recently developed even to detect the ochratoxin A mycotoxin in cereal samples (Chen S. et al., 2024) is a novel and promising alternative to the conventional methods for the detection and identification of *Alternaria*. Traditional morphological identification of *Alternaria* isolates includes characterizing colony and conidia morphology, such as shape, color, size, and other characteristics. *Alternaria* species represent distinct characteristics, classified into six groups and used to differentiate and characterize *Alternaria* isolates (Simmons and Roberts, 1993; Simmons, 2007; Poursafar et al., 2018). Phylogeny studies are performed based on different genetic loci, including plasma membrane ATPase, calmodulin, and glyceraldehyde-3-phosphate dehydrogenase (GAPDH) (Lawrence et al., 2013, 2016; Woudenberg et al., 2013) while metabolites profiling method was also able to differentiate certain *Alternaria* sections (Patriarca et al., 2019). Conventional PCR based on the ITS region was developed to detect *Alternaria* spp. in crop samples as well, including wheat, barley, maize, sorghum, mustard, cowpea, and other crops (Zur et al., 2002; Chakdar et al., 2019). PCR-based methods, including Sequence-Related Amplified Polymorphism (SRAP) (Castañares et al., 2023) and inter-primer binding site (iPBS) DNA profiling technique (Turzhanova et al., 2020), as well as LAMP-based detection (Yang et al., 2019), were also developed for *Alternaria* identification.

CRISPR/Cas12a advances traditional identification methods by potentially applying directly on-site diagnostics with high specificity and sensitivity. Our results confirm the application potential of the method to identify *Alternaria* with remarkable sensitivity down to 100 copies and high specificity toward *Alternaria* isolates. In the specificity assays, we included different fungal species selected based on how frequently they are reported as isolated from wheat as well as how dominant they are on wheat grain fungal population and production worldwide, including Kazakhstan. For example, the tested *B. sorokiniana*, *F. acuminatum*, and *F. equiseti*, species were reported as the most predominant fungal species among 1,221 strains (44.8, 20.39, and 10.16%, respectively) isolated from wheat collected in different wheat-sowing regions in Kazakhstan (Bozoğlu et al., 2022). Furthermore, *F. acuminatum* is considered an emerging dominant pathogen in North America, Brazil, and Southern Europe (Laraba et al., 2022), while *B. sorokiniana* and *F. equiseti* are also known as widely distributed fungal pathogens frequently infecting wheat and other cereal crops worldwide (Özer et al., 2020; Shrestha et al., 2021; El-Morsy et al., 2023; Roy et al., 2023). *N. oryzae* is also considered an emerging pathogen (Sha et al., 2023), while its first detection in Kazakhstan on wheat was documented in 2016 (Eken et al., 2016). None of these isolates exhibited a reaction either in RPA amplification itself or in fluorescence assays with the CRISP/Cas12a-based method, suggesting the high *Alternaria*-specificity of the proposed method.

The high specificity of the method is based not only on the selection of proper RPA primers but also on the specificity of gRNA, enabling precise recognition of the PAM site in the target genetic locus and triggering subsequent *cis* and *trans*-activities of Cas12a. As part of the method development, we examined further the potential of our novel Cas effector homolog, MbCas12a from *M. bovis*. Previously, it was also successfully employed to detect *Staphylococcus aureus* and *E. coli*, the causing agents of bovine mastitis in milk samples (Shaizadinova et al., 2023; Amanzholova et al., 2024). Effective validation of the MbCas12a-based method on the plant fungal pathogen *Alternaria* also confirms the practical utilization of our enzyme as a suitable alternative to the previously reported available analogs in CRISPR/Cas diagnostics. Sufficient *cis*-activity of MbCas12a was also demonstrated toward precise cleavage of the target 177-bp long β-tubulin sequence up to 93 and 84 bp fragments. MbCas12a employs different PAM sequences, such as TTTA, TCTA, TTCA, TCCA, CTTA, CCTA, and CCCA (Shaizadinova et al., 2023). In addition, in the present studies, we successfully applied the GTTG region in the sequence of the β-tubulin.

Our studies centered on the β-tubulin gene as a detection target because it is highly conserved and offers unique, well-suited regions to construct specific primers (Nahimana et al., 2000; Kumar et al., 2013). The homology of the selected region for crRNA and RPA primers in β-tubulin in different *Alternaria* spp. and no such homology in other wheat fungal pathogenic species (Supplementary Figure S9) confirms the sustainability of this gene for the proposed method. Consequently, β-tubulin is a widely used molecular marker for detecting and identifying *Alternaria* spp. in different crops and plants. For example, it was used for the identification of *A. alternata* in loquat and olive trees (Basım et al., 2017; Abbas et al., 2020). β-tubulin was also utilized as a molecular marker to identify *Alternaria* spp. in trees, including *Haloxylon aphyllum*, *Tamarix hispida*, and *Tamarix ramossisima* (Sherimbetov et al., 2024). Moreover, β-tubulin was used for the identification of different *Alternaria* pathogenic species, including *A. alternata*, *A. tenuissima*, *A. arborescens*, *A. brassicicola*, and *A. japonica* in vegetables (Siciliano et al., 2017). Specifically designed primers targeted β-tubulin amplified *A. solani* only while no amplification was detected in 13 closely related species (Kumar et al., 2013). It was also previously applied for *A. alternata* identification (Kordalewska et al., 2015) in which no amplification was observed in different fungi, molds, and yeast-like fungi, including *Fusarium*, *Ulocladium*, *Geotrichum*, and others, collected from various sources, supporting its suitability for this study (Kordalewska et al., 2015). Along with the gpd and calmodulin genes, all 74 *Alternaria* strains isolated from wheat kernels collected from 36 different durum wheat fields were amplified with the β-tubulin gene (Masiello et al., 2022). Furthermore, β-tubulin was also used in the CRISPR/Cas12a technology to detect and identify the causing agents of wheat rust *P. striiformis* f. sp. *Tritici* (Sánchez et al., 2022). The CRISPR/Cas12a method was also successfully employed for the detection of many other wheat pathogens, such as *F. asiaticum* (Zhang et al., 2023), *F. graminearum* (Mu et al., 2022), and *Magnaporthe oryzae Triticum* (Kang et al., 2021; Sánchez et al., 2022). Regarding *Alternaria* detection in crop samples by CRISPR/Ca12a method, a set of subsequent studies using different amplification methods (PCR, RPA, and RCA) and visualization tools (photothermal platform, G-quadruplex) was efficiently performed to detect *Alternaria* spp. associated with citrus, tomato, and apple diseases (Liu et al., 2022, 2023; Ma et al., 2024). Also, the potato-devastating pathogen *A. solani* was targeted with CRISPR/Cas12a method based on the 5.8S rRNA gene and LAMP amplification (Guo H. et al., 2023). However, to our knowledge, no report about the CRISPR/Cas12a-RPA-based identification of *Alternaria* spp. in wheat samples has been reported so far. In addition to the shown sensitivity and specificity, we also integrated a fluorescence assay to demonstrate the feasibility of CRISPR/Cas12a as the field-deployable diagnostic tool. These assays do not require ancillary equipment, suggesting the potential of the proposed method for on-site diagnostics. These method characteristics could facilitate its potential implementation to detect, control, and monitor *Alternaria* spp., making protection management easier and thereby reducing potential crop losses, which eventually contribute to the sustainability of food security.

The CRISPR/Cas12a-based detection method’s rapidity, low equipment requirement, and cost-effectiveness make it particularly advantageous for use in resource-limited settings. These characteristics could significantly enhance early pathogen detection in rural or field-based agricultural practices, where access to advanced laboratory facilities is limited. Early and accurate detection of *Alternaria* and other phytopathogens can enable timely intervention, minimizing crop loss and safeguarding food security. The assay could be further adapted for field use with lateral-flow dipsticks, allowing colorimetric readout and enhancing accessibility in resource-limited settings. Similar lateral-flow adaptations were already reported for CRISPR-based assays (Kaminski et al., 2021; Tanny et al., 2023) and adapted for specific plant pathogenic species (Kang et al., 2021; Lei et al., 2022). Further assays could explore lyophilized reagent formats or stabilizing agents to maintain enzyme functionality at ambient temperature to enhance field application of the CRISPR/Cas12a method.

## 5 Conclusion

Based on our findings, the CRISPR/Cas12a method employing RPA amplification was successfully applied to detect *Alternaria* strains isolated from wheat grain samples. The technique centered on the β-tubulin gene demonstrated high specificity for *Alternaria* strains and remarkable sensitivity toward fungal DNA. The results can be visualized using the fluorescence method, facilitating the field-deployable implementation of the method. Additionally, the obtained results expand the application of MbCas12a, which consequently broadens the spectrum of the Cas12a proteins applied in the CRISPR/Cas12a technology. Together, these results demonstrate that the proposed method can be used to detect and identify *Alternaria* isolates in wheat samples with high accuracy, sensitivity, and specificity, whether in a certified laboratory or for on-site diagnostics.

## Data Availability

The datasets presented in this study can be found in online repositories. The names of the repository/repositories and accession number(s) can be found in the article/Supplementary material.

## Footnotes

^1^ https://posit.co/
