# Supplementary material for: CRISPR/Cas12a-based method coupled with isothermal amplification to identify Alternaria spp. isolated from wheat grain samples
Source: Front Microbiol. 2025 Jan 15;15:1468336. doi: 10.3389/fmicb.2024.1468336 (PMC11775006; doi:10.3389/fmicb.2024.1468336)
Supplement: Supplementary file 1 [file Data_Sheet_1.pdf]

Supplementary Material

1 Supplementary Data

Supplementary Material includes full scans of the entire original gels of all corresponding figures presented in the manuscript (as requested by Frontiers Initial Validation Team). Certain explanatory comments to original gels are given in italic font below the figure legend.

2 Supplementary Figures and Tables

2.1 Supplementary Figures

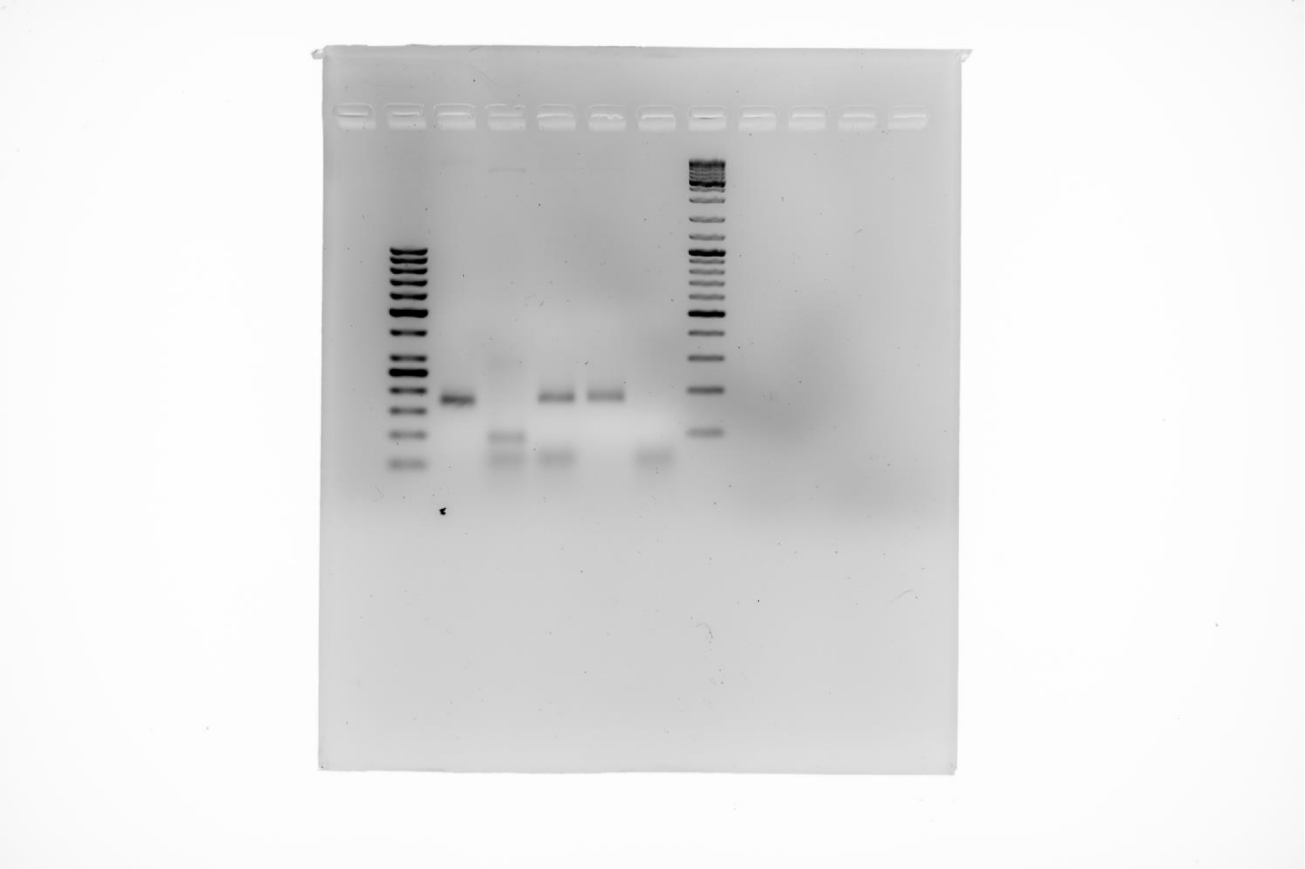

Supplementary Figure 1. Original gel of Figure 3A

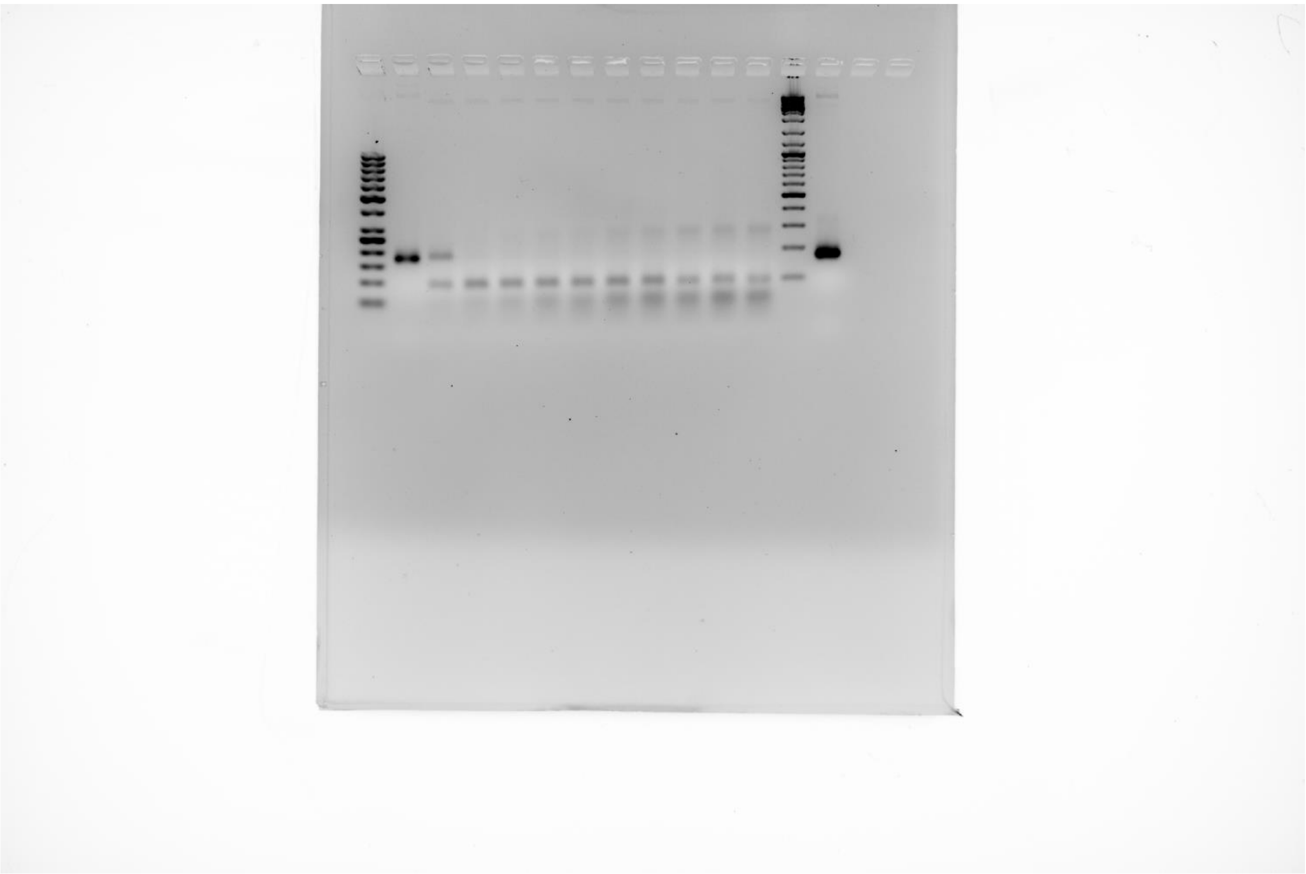

Supplementary Figure 2. Original gel of Figure 3B

A single sample after the second marker is an additional PCR product added in gel without any reaction (not shown in the manuscript to avoid redundant information). The difference is it was mixed with higher amount of dye to determine optimal dye concentration.

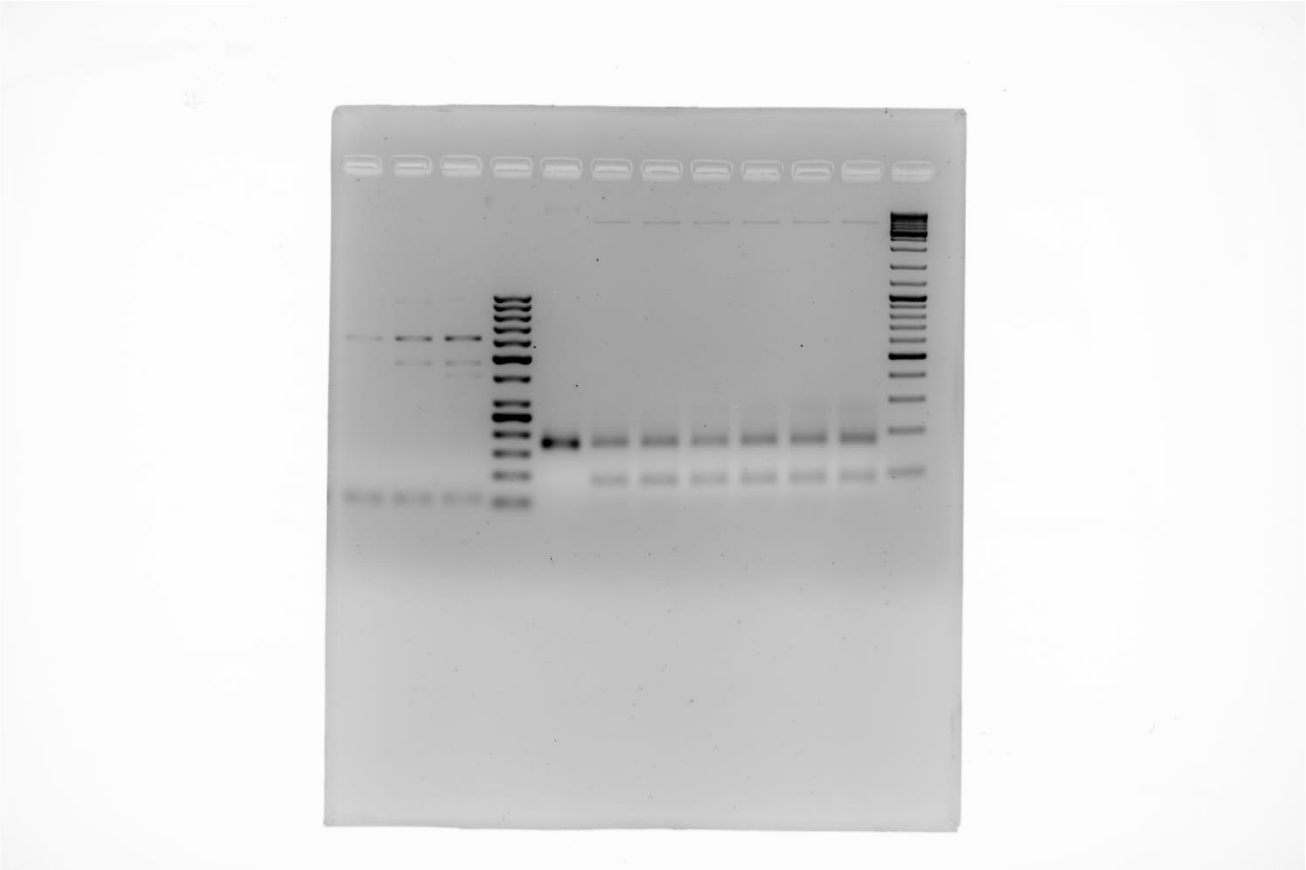

Supplementary Figure 3. Original gel of Figure 4A

Three samples on the left next to the first marker are samples from another separate test. They were added to run samples together in one gel to preserve materials and minimize ethidium bromide consumption.

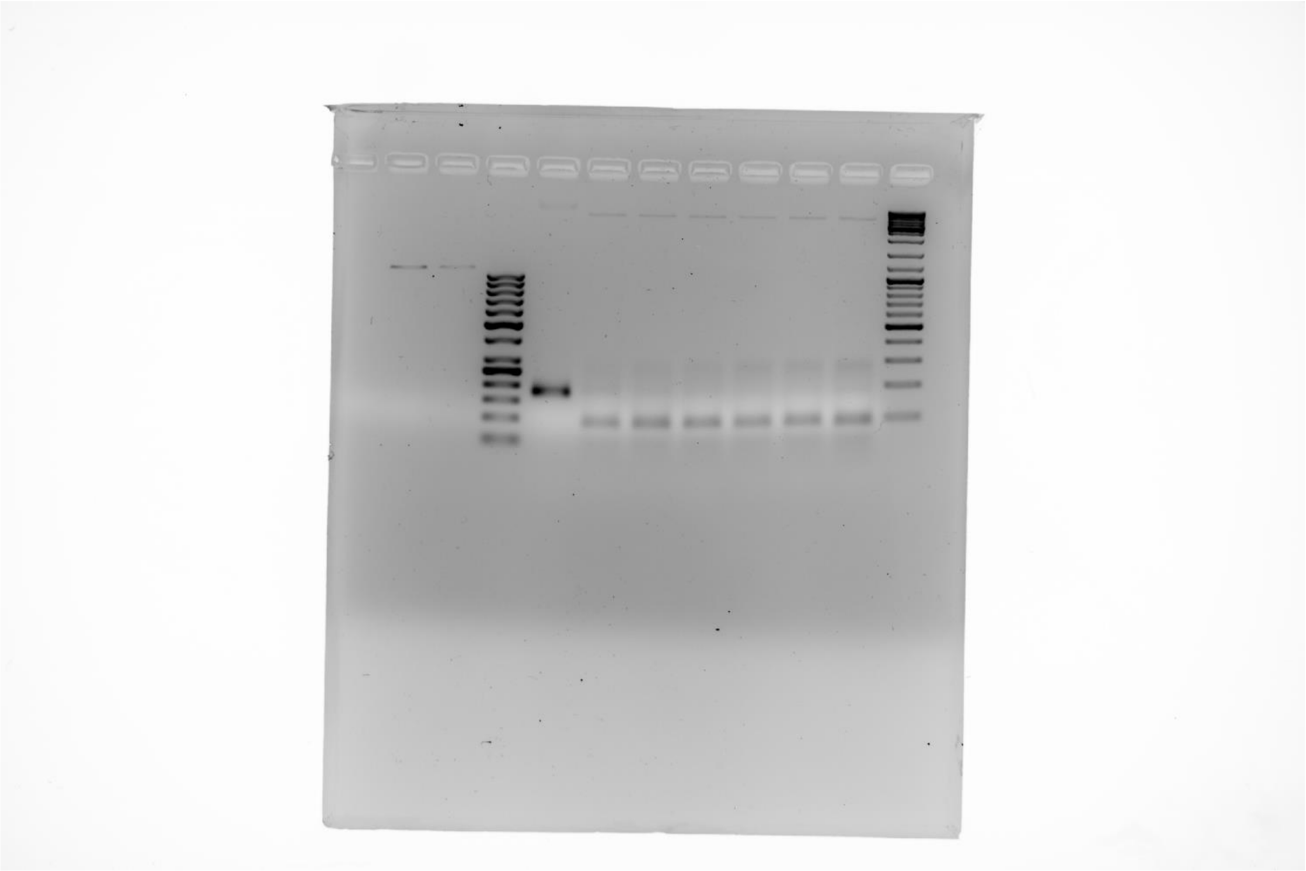

Supplementary Figure 4. Original gel of Figure 4B

Two samples on the left next to the first marker are samples from another separate test. They were added to run samples together in one gel to preserve materials and minimize ethidium bromide consumption.

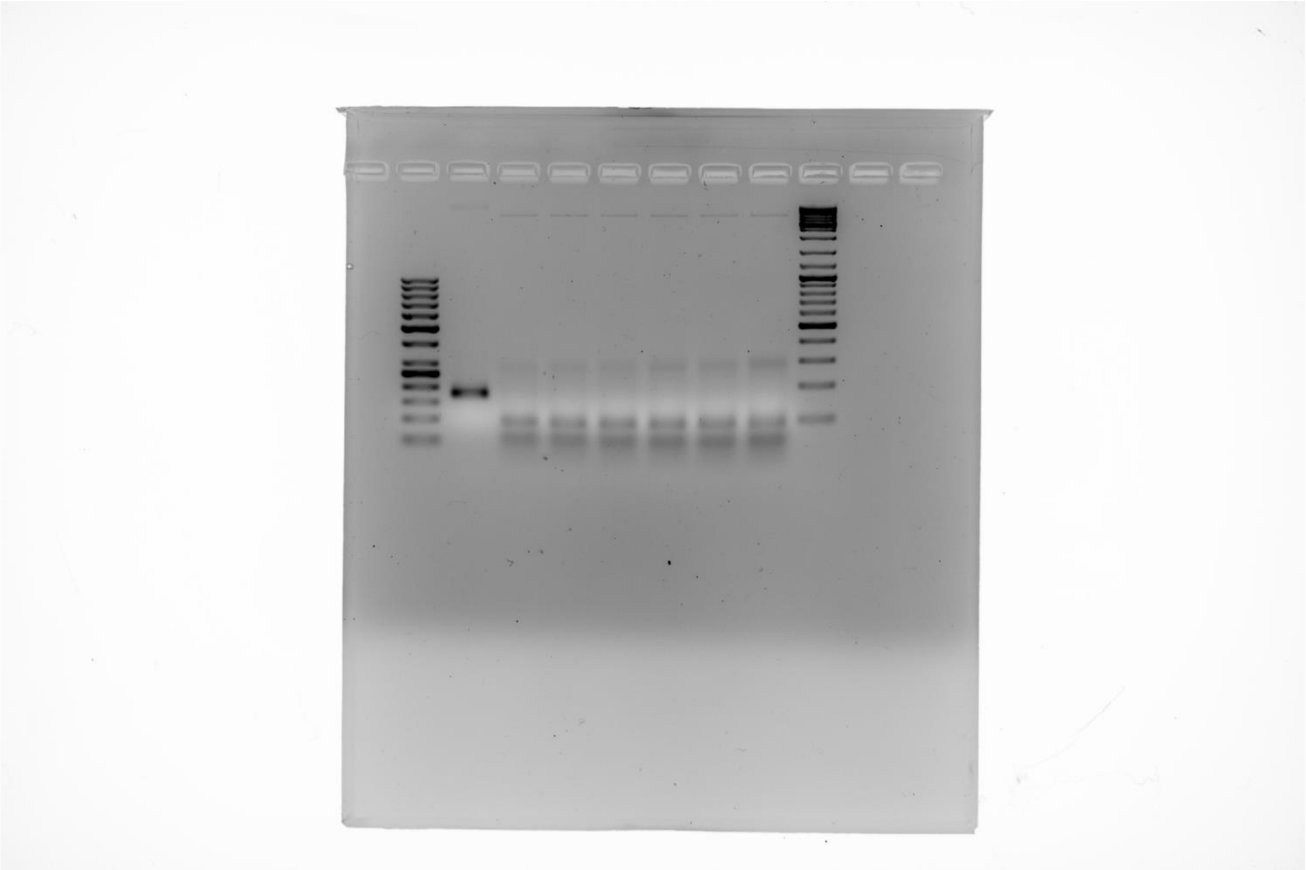

Supplementary Figure 5. Original gel of Figure 4C

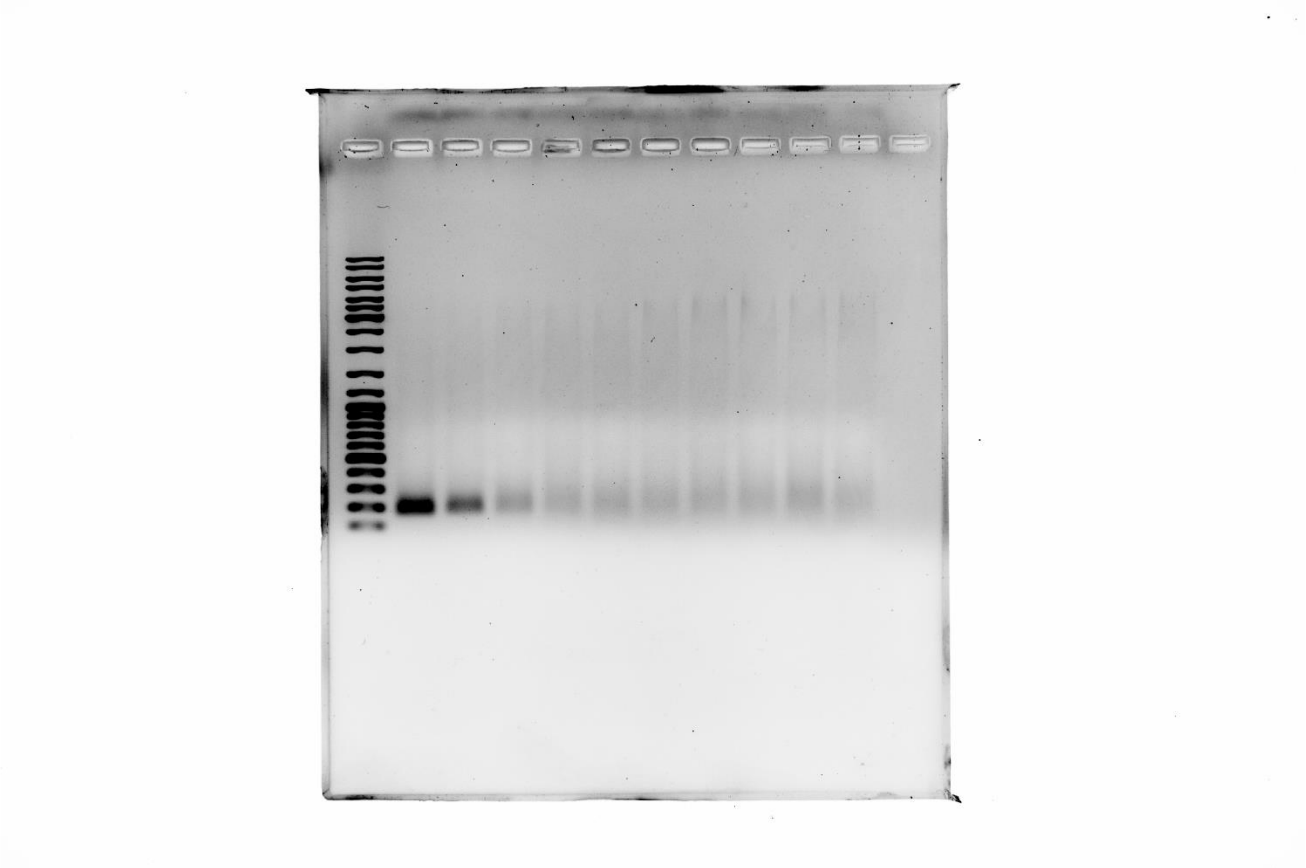

Supplementary Figure 6. Original gel of Figure 5B

Here: samples in rows 1-7 are 0, 10^{-1}*,* 10^{-2}*,* 10^{-3}*,* 10^{-4}*,* 10^{-5}*,* 10^{-6} *as stated in the manuscript. Samples 8-9 are DNA copies at* 10^{-7} *and* 10^{-8} *or 0.12 and 0.012 DNA copies, respectively. They were tested but not shown in the manuscript to avoid redundant information. The row 10 is water (negative control), stated in the manuscript as sample 8. The last sample is empty.*

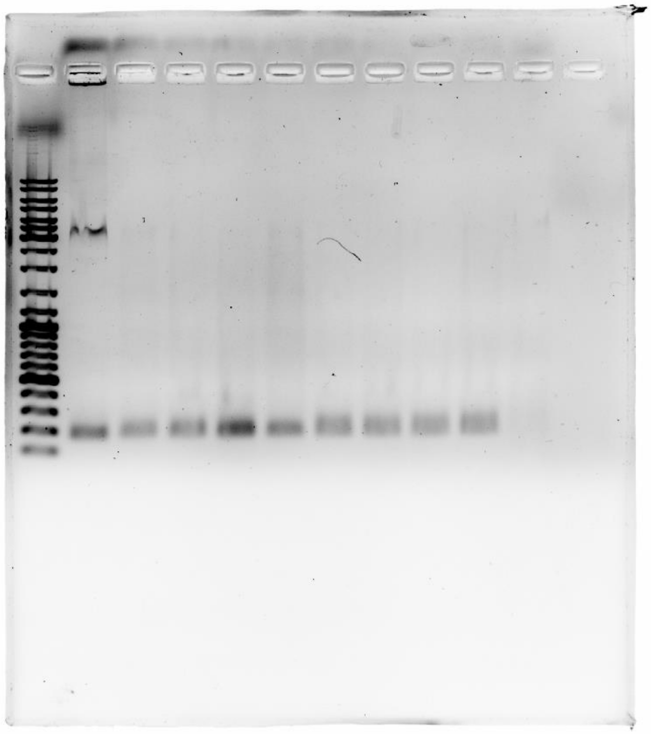

Supplementary Figure 7. Original gel of Figure 6A

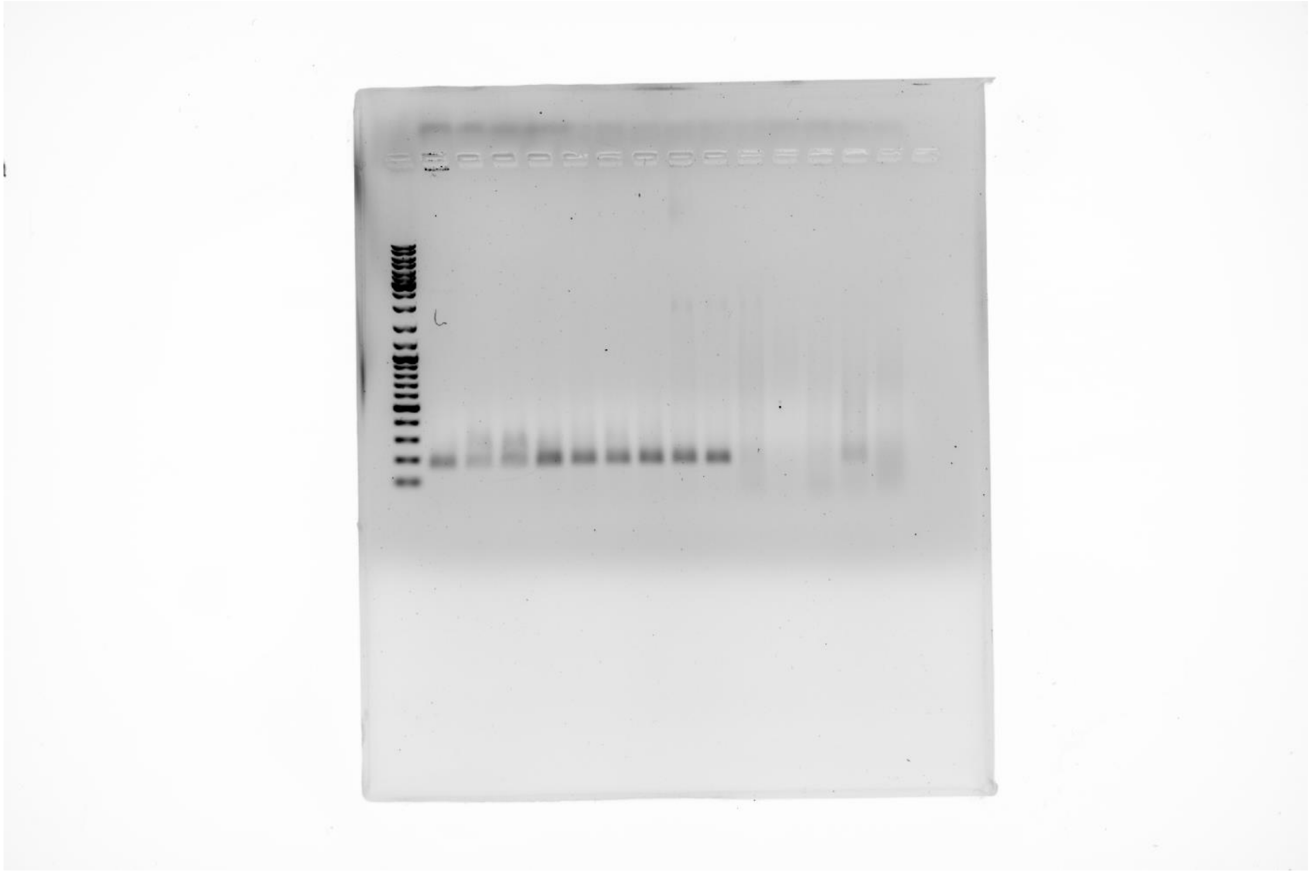

Supplementary Figure 8. Original gel of Figure 7A

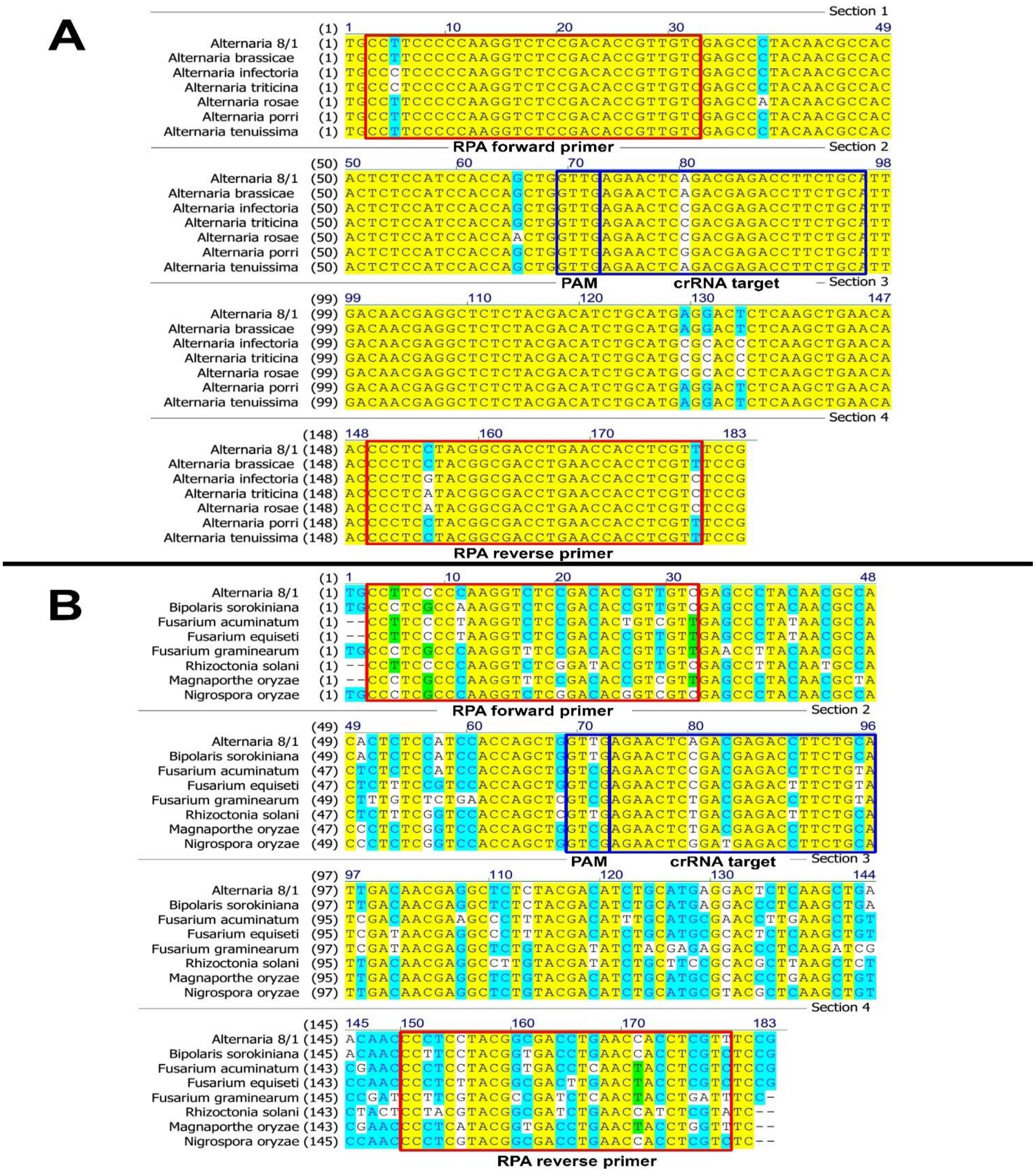

Supplementary Figure 9. Multiple alignments of β-tubulin of *Alternaria* species and different fungal plant pathogenic species. **A** - *Alternaria* species commonly infected wheat, **B** - other wheat pathogenic fungal species. *Alternaria* 8/1 strain is sequenced β-tubulin used as positive control in the study, the remaining sequences are retrieved from NCBI under corresponding references: *Alternaria brassicae* CP145300.1, *Alternaria infectoria* ASM2404317v1, *Alternaria triticina* DAOMC238612_v1, *Alternaria rosae* XM_046171018.1, *Alternaria porri* RDWU_Aporii_p.ctg, *Alternaria tenuissima* KT920416.1, *Cochliobolus sativus* (*Bipolaris sorokiniana*) KB445648.1, *Fusarium acuminatum* CP151260.1, *Fusarium equiseti* ASM331317v1, *Fusarium graminearum* CM000577.1, *Rhizoctonia solani* CP059666.1, *Magnaporthe oryzae* CM001235.1, *Nigrospora oryzae* ASM1675884v1.

2.2 Supplementary Table

Supplementary Table 1. Comparison of different detection assays for pathogen identification

Assay type	Methodology	Time	Specificity	Sensitivity	Cost	Reference
Culture-based methods and microscopy	Identification of pathogens on selective media or based on morphological characteristics	24–72 hours	Low to moderate	Low to moderate	Low to moderate	Zhao et al. (2014)
Biochemical assays	Biochemical reaction-based identification of metabolic properties	Minutes-hours	Moderate to high	Low to moderate	Low to moderate	Franco-Duarte et al. (2019)
Immunological-based diagnostic methods	Antibody-based detection and quantification of antigens	2–3 hours	High	High	Moderate	Hariharan and Prasannath (2021)
PCR	Amplification of DNA target using specific primers in a thermocycler	2–4 hours	Very high	Very high	Moderate to high	Ray et al. (2017)
Real-time PCR	Quantitative DNA amplification in real-time using fluorescent probes	1–3 hours	Very high	Very high	High	Aslam et al. (2017)
Isothermal amplification (LAMP, RPA)	Amplifies DNA/RNA at a low constant temperature, suitable for a field-portable setup	30–60 min	High	High	Moderate	Notomi et al. (2000); Piepenburg et al. (2006)
CRISPR-based diagnostics	Uses CRISPR-associated proteins with crRNAs to detect target DNA	20-60 min	Very high	Very high	Low	Kaminski et al. (2021)

References

Aslam, S., Tahir, A., Aslam, M. F., Alam, M. W., Shedayi, A. A., and Sadia, S. (2017). Recent advances in molecular techniques for the identification of phytopathogenic fungi – a mini review. *J. Plant Interact.* 12, 493–504. doi: 10.1080/17429145.2017.1397205.

Franco-Duarte R., Černáková L., Kadam S., Kaushik K., Salehi B., Bevilacqua A., et al. (2019). Advances in chemical and biological methods to identify microorganisms—from past to present. *Microorganisms* 7:130. doi: 10.3390/microorganisms7050130

Hariharan, G., and Prasannath, K. (2021). Recent advances in molecular diagnostics of fungal plant pathogens: a mini review. *Front. Cell. Infect. Microbiol.* 10, 600234. doi: 10.3389/fcimb.2020.600234.

Kaminski, M. M., Abudayyeh, O. O., Gootenberg, J. S., Zhang, F., and Collins, J. J. (2021). CRISPR-based diagnostics. *Nat. Biomed. Eng.* 5, 643–656. doi: 10.1038/s41551-021-00760-7.

Notomi, T., Okayama, H., Masubuchi, H., Yonekawa, T., Watanabe, K., Amino, N., et al. (2000). Loop-mediated isothermal amplification of DNA. *Nucleic Acids Res.* 28, e63–e63. doi: 10.1093/nar/28.12.e63.

Piepenburg, O., Williams, C. H., Stemple, D. L., and Armes, N. A. (2006). DNA detection using recombination proteins. *PLOS Biol.* 4, e204. doi: 10.1371/journal.pbio.0040204.

Ray, M., Ray, A., Dash, S., Mishra, A., Achary, K. G., Nayak, S., et al. (2017). Fungal disease detection in plants: Traditional assays, novel diagnostic techniques and biosensors. *Biosens. Bioelectron.* 87, 708–723. doi: 10.1016/j.bios.2016.09.032.

Zhao, X., Lin, C.-W., Wang, J., and Oh, D. H. (2014). Advances in rapid detection methods for foodborne pathogens. *J. Microbiol. Biotechnol.* 24, 297–312. doi: 10.4014/jmb.1310.10013.
